# Ultrasonographic Algorithm for the Assessment of Sentinel Lymph Nodes That Drain the Mammary Carcinomas in Female Dogs

**DOI:** 10.3390/ani10122366

**Published:** 2020-12-10

**Authors:** Florin Stan, Alexandru Gudea, Aurel Damian, Adrian Florin Gal, Ionel Papuc, Alexandru Raul Pop, Cristian Martonos

**Affiliations:** 1 Department of Comparative Anatomy, Faculty of Veterinary Medicine, University of Agricultural Sciences and Veterinary Medicine, 3-5 Manastur Street, 400372 Cluj Napoca, Romania; alexandru.gudea@usamvcluj.ro (A.G.); aurel.damian@usamvcluj.ro (A.D.); cristian.martonos@usamvcluj.ro (C.M.); 2 Department of Cell Biology, Histology and Embryology, Faculty of Veterinary Medicine, University of Agricultural Sciences and Veterinary Medicine, 3-5 Manastur Street, 400372 Cluj Napoca, Romania; adrian.gal@usamvcluj.ro; 3 Department of Semiology and Medical Imaging, Faculty of Veterinary Medicine, University of Agricultural Sciences and Veterinary Medicine, 3-5 Manastur Street, 400372 Cluj Napoca, Romania; ionel.papuc@usamvcluj.ro; 4 Department of Reproduction, Obstetrics and Reproductive Pathology, Biotechnologies in Reproduction, Faculty of Veterinary Medicine, University of Agricultural Sciences and Veterinary Medicine, 3-5 Manastur Street, 400372 Cluj Napoca, Romania; alexandru.pop@usamvcluj.ro

**Keywords:** sentinel lymph node, canine mammary tumor, staging, ultrasonography, non-invasive

## Abstract

**Simple Summary:**

Mammary neoplasms are one of the most common oncological diseases diagnosed in female dogs. Their staging also involves the evaluation of the sentinel lymph nodes that drain these tumors. Since non-invasive diagnosis is an important requirement in both human and veterinary medicine, by using simple and available ultrasound techniques, our study proposes a non-invasive assessment of the status of sentinel lymph nodes of the tumoral mammary glands. The algorithm consists of B-mode ultrasound, Doppler technique, contrast-enhanced ultrasound (CEUS), and real-time elastography. Diagnostic performance was established for each technique. The highest accuracy in identifying metastases in sentinel lymph nodes was given by the elasticity score followed by the short/long-axis ratio and resistivity index. CEUS had the same accuracy as the Doppler examination. By assigning a score to each parameter of the mentioned techniques and summing up these scores, we obtained a high accuracy of metastasis detection in sentinel lymph nodes (92.2%). This algorithm for examining the status of sentinel lymph nodes that uses simple and widely available techniques in the current practice can be extremely useful to practitioners for staging mammary malignant tumors in female dogs, leading to the right treatment decision.

**Abstract:**

The status of sentinel lymph nodes (SLNs) is decisive in staging, prognosis, and therapeutic approach. Using an ultrasonographic examination algorithm composed of B-mode, Doppler technique, contrast-enhanced ultrasound (CEUS) and elastography, this study aimed to determine the diagnostic performance of the four techniques compared to histopathological examination. 96 SLNs belonging to 71 female dogs with mammary gland carcinomas were examined. After examinations, mastectomy and lymphadenectomy were performed. Histopathological examination confirmed the presence of metastases in 54 SLNs. The elasticity score had the highest accuracy—89.71%, identifying metastases in SLNs with 88.9.9% sensitivity (SE) and 90.5% specificity (SP), ROC analysis providing excellent results. The S/L (short axis/long axis) ratio showed 83.3% SE and 78.6% SP as a predictor of the presence of metastases in SLN having a good accuracy of 81.2%. On Doppler examination, the resistivity index(RI) showed good accuracy of 80% in characterizing lymph nodes with metastases versus unaffected ones; the same results being obtained by CEUS examination. By assigning to each ultrasonographic parameter a score (0 or 1) and summing up the scores of the four techniques, we obtained the best diagnostic performance in identifying lymph node metastases with 92.2% accuracy. In conclusion, the use of the presented algorithm provides the best identification of metastases in SLNs, helping in mammary carcinoma staging and appropriate therapeutic management.

## 1. Introduction

It is well known that the status of sentinel lymph nodes in mammary carcinomas in both women and female dogs is a prognostic factor of the disease and decisively influences the therapeutic approach. The correlations between the two species are justified because the spontaneous mammary tumors in dogs have biological and histopathological common appearances with those of the woman, being valuable experimental models in the study of breast cancers [1,2]. Another worth considering aspect is the similar mechanism of metastasis, through the lymphatic pathways, initially in the regional lymph nodes, for most of the carcinomas. Furthermore, tumor staging is dependent on the presence or absence of tumor invasion in these lymph nodes [3,4]. As a first instance, the lymph node that drains the tumor must be identified and then investigated to determine its status-unaffected or affected/metastatic. In breast cancer in women, there are standard protocols for the identification of SLNs (sentinel lymph nodes) by lymphoscintigraphy after peritumoral or subareolar injection of 99mTc followed by dye injection. [5,6,7]. The deficiency of this protocol is given by the side effects of Tc radioactivity, relatively high costs, and dye-related side effects (possible allergic reactions, local necrosis, and the inability to perform the biopsy technique in real-time). The newly developed technique of localization of sentinel lymph nodes (SLN), near-infrared fluorescence imaging, which used indocyanine green (ICG) as a mapping agent is successfully used in women [8,9] and is being investigated as a possible technique of mapping the SLNs of mammary tumors in bitches [10]. However, the best results in the identification and evaluation of SLNs are obtained by CT lymphography [11,12,13], Positron Emission Tomography–Computed Tomography (PET/CT), [14,15], or MRI [16], both in woman and bitch. Currently, ultrasonography is the first method of evaluation, being noninvasive, widely available, and easy to perform in clinical practice.

Using gray-scale ultrasonography, the criteria for differentiating benign from malignant lymph nodes refer to size, shape, nodal border, echogenicity, hilum appearance, calcification, or necrosis, alongside the appearance of vascularization at Doppler examination. It was stated that the malignant lymph nodes have a rounded shape, the S/L ratio is over 0.5, (short axis/long axis ratio), a hypoechoic pattern, absence of echogenic hilum, sharp borders, and chaotic, peripheral or mixed vascularization [17,18,19,20]. However, these findings are not significant for accurate differentiation of metastatic lymph nodes. Contrast-enhanced ultrasound (CEUS), a recent, novel technique for evaluating tissue perfusion in real time, became widely used in the evaluation of SLN in breast cancer in women [21,22,23,24]. This technique received attention in the evaluation of SLNs draining mammary tumors in bitches [25,26] or healthy draining mammary lymph nodes [10,27]. The accuracy of CEUS in identifying SLNs is limited if the contrast agent is injected intravenously, as there is a risk of evaluating a regional lymph node that does not drain the tumor. Another limitation of the lymph node assessment by CEUS lies in the fact that this method evaluates only the lymph node that has been identified presumed to be sentinel (because it is part of the lymph nodes that are anatomically present in the region of interest and usually drain the basin in which the tumor is located). With this method, the lymphatic drainage that can be followed at a given time is generally only one, meaning that different drainage basins are unlikely to be monitored at the same time. By intradermal or peritumoral injection, this deficiency is removed as much as possible.

Another recent technique that relevantly assesses SLNs is elastography. Considering that tumor infiltration increases the SLNs stiffness, this technique is increasingly applicable in animals lymph node evaluation. [28,29,30]. There are two main types of elastography—real-time elastography (or strain elastography) and share wave elastography (SWE) [31,32]. Real-time elastography qualitatively assess the degree of stiffness of the lymph node in the form of elasticity scores and quantitatively evaluates the strain ratio. SWE provides a quantitative value of tissue stiffness expressed in shear wave speed in meters/second or converted by the software and expressed in kPa requiring special modules incorporated in the ultrasound device.

The presence of metastases in the SLNs can be confirmed only after the true sentinel lymph node is identified and assessed. It may be possible that SLN is not the closest lymph node from the tumor drainage basin. This is due either to obstruction of the lymphatic pathways with tumor thrombi, or cause of an increased peritumoral lymphangiogenesis that changes the lymphatic pathway to another lymph node [33,34,35]. It is well-known that healthy mammary glands drain into two main lymph centers—axillary, through proper and accessory LNs, respectively the inguinofemoral lymph center through superficial inguinal LNs [36,37]. In the case of mammary neoplasia, the lymphatic drainage is altered, as other lymph centers might be involved—for example, the ventral thoracic lymph center through the cranial sternal lymph nodes or even the superficial cervical lymph center through the ventral superficial cervical lymph nodes [33,34].

Thus, it is imperative to identify and evaluate with the highest accuracy the real and corresponding SLN of the tumor drainage basin. Considering the above-mentioned facts and having as investigation methods the simple and available ultrasound methods (namely grayscale US, Doppler technique, CEUS, and elastography), this study aims to provide a non-invasive algorithm for SLNs evaluation. The accuracy of each method in differentiating between benign and metastatic SLN will be established having as reference the histopathological examination.

## 2. Materials and Methods

The study protocol and design complied with the guidelines of the Romanian national legislations, according to European Union standards regarding animals involved in clinical studies. The Bioethics Committee of the University of Agricultural Sciences and Veterinary Medicine approved the study (approval no. 79/17.11.2017). Informed consent was signed by the owners of animals included in the study.

A prospective cohort study was conducted including 71 female dogs of various pure and mixed breeds, diagnosed with malignant mammary tumors of the cranial thoracic (T1), cranial abdominal (A1), caudal abdominal (A2), and inguinal (I) mammary glands, histopathologically confirmed by biopsy. The subjects were chosen from patients presented for examination at the university hospital due to the presence of one or more mammary tumors. To be eligible for the study, the subjects were supposed to meet the following criteria: no previous therapy for the mammary tumor, no evidence of distant metastasis revealed by three-view thoracic radiographs and abdominal ultrasound, absence of another serious illness (severe heart and respiratory conditions, cachexia or other types of neoplasms, including lymphomas) and absence of pregnancy.

Clinical examination, complete blood count, serum biochemistry, and urine analysis were performed before the examinations. All animals were examined using an algorithm composed of greyscale ultrasound, Doppler technique, contrast-enhanced ultrasound, and real-time elastography. The dogs were examined in dorsal recumbency being closely monitored throughout the examinations. Sedation during examinations was achieved by the IV administration of acepromazine (Sedam, Pasteur Romania) at 0.2–0.3 mg/kg BW.

The procedure started with low doses of acepromazine being augmented during the examination protocol in cases that have required a much longer examination time. This approach was also a consequence of some cited effects of anesthetics on CEUS parameters that have been influencing the perfusion of different organs in dogs and cats [38,39].

### 2.1. Gray Scale Examination

The included subjects were examined with a Logiq E9 GE (MEDIST Imaging, GE Healthcare, Romania) ultrasound device or a Phillips IU-22 XMatrix Diamond Select (Danson Medicine, Bucuresti, Romania) device. For B–Mode examination a high-frequency linear probe, 6–15 MHz was used. A single experienced sonographer performed the examinations.

The study was conducted according to ARRIVE guidelines [40]. The examiner was unaware of the histopathological result of the SLNs throughout the ultrasonographic evaluation. On greyscale ultrasound, the following parameters were recorded: value (in cm) of the short and long axis (for the short/long ratio calculation), the internal structure (echostructure) classified as homogeneous or inhomogeneous, the echogenicity of the lymph node classified as hypoechoic, isoechoic or hyperechoic, hilum tissue definition recorded as present or absent/invisible and the capsule pattern recorded as well-defined or ill-defined. The measurements were made, having as reference a series of known reports and procedures in humans and animals [41,42,43].

### 2.2. Doppler US Examination

Doppler US examination used pulse repetition frequency 350 Hz and wall filter 45 Hz. Color Doppler technique (with pulse repetition frequency kept low—to maximize vessel detection—and the angle between the Doppler beam and long axis of the vessel being kept under 60°) studied the presence and distribution of the vascular signal. The region of interest was carefully examined without exerting pressure on the lymph node as compression may obliterate the vascular signal.

Four patterns of vascular signal localization were recorded: hilar pattern, peripheral pattern, mixed vascularisation (with both hilar and peripheral pattern), or absence of vascular signal. We used the same classification as in reports on vascularization found in mammary gland tumors in female dogs [43,44] and lymph nodes draining various tumors [42]. Regarding the type and distribution of vessels, two patterns were defined: ordered or chaotic vascularisation [41]. Pulse wave Doppler analysis was used for vascular indices assessment. After the best color signals were obtained, the spectral gate was placed on the main artery of the node which showed the fastest arterial signal.

Resistive index (RI), pulsatility index (PI), peak systolic velocity (PSV) and end-diastolic velocity (EDV) were measured using integrated software. The RI was calculated as follows: peak systolic velocity–end-diastolic velocity/peak systolic velocity. PI was calculated as follows: peak systolic velocity–end-diastolic velocity/time-averaged maximum velocity [28,45].

Two consecutive measurements were made and the average value for RI and PI was calculated for each lymph node.

### 2.3. CEUS Examination

CEUS examination used a 3–9 MHz linear transducer (range of gain: 86–90%, compression 38, low mechanical index of 0.07, and dedicated contrast software). In each subject, using a 2 mL syringe with a 26G needle, peritumoral administration of 0.5 mL SonoVue (Bracco Imaging SpA, Milan Italy), was performed in each point, at four symmetrical points around the tumor, in the subcutaneous cellular tissue, followed by gentle massage of the region. The microbubble contrast agent was reconstituted in 2 mL of saline water, containing 1 × 109 microbubbles/mL as previously reported [46].

The lymphatic channels were traced on contrast pulse sequencing until they reached the corresponding lymph node, which has been assumed to be the sentinel lymph node [26]. Three patterns of enhancement were defined: intense homogeneous pattern, inhomogeneous pattern with perfusion defects, and no enhancement [47].

The contrast transit times were recorded as perfusion phases, (wash-in time and wash-out time, in seconds). We defined wash-in time as the period from time zero (after the SonoVue injection) to first cortical enhancement, ending by complete medullary enhancement of lymph node parenchyma. Wash-out time was defined as the time from the visually-assessed first decrease in medullary enhancement followed by a slower cortical enhancement (in the whole or in a part of the lymph node) resulting in hypoenhancement of the lymph node [44,48].

### 2.4. Real-Time Elastography (RTE)

Real time elastography was performed with a high-resolution 7–18 MHz real-time linear-array transducer and devices elastographic software. Before the RTE, conventional US was performed to obtain the most appropriate information of each SLN. The area where the lymph node was identified by CEUS was also considered. Then, the elastogram image was displayed along the B-mode image in a two-panel appearance. The region of interest (ROI) was established to encompass the entire lymph node and approximately the same part of adjacent tissue (excluding tissues like bone and blood vessels that may affect stiffness assessment). Light pressure along the radiation axis, followed by decompression was made until the same size and color images in sequential frames were obtained [31,49]. The direction of compression was upwards and downwards.

According to the color-graphic representation (in which blue indicated stiff, green and yellow indicated intermediate stiffness and red indicated soft), the lymph nodes were categorized using a five-point elasticity score proposed by Alam and collaborators (Table 1) [49]. Three to five measurements were made for each lymph node. The image that met at least twice the same classification was chosen for assigning the final score.

The female dogs were carefully monitored for 24 h after the ultrasonographic examination.

All examinations were supervised by two experienced practitioners—one with experience in human imaging and the second in veterinary imaging.

Mastectomy and ipsilateral lymphadenectomy were performed in all subjects. Before surgery, lymphatic mapping was performed. In this respect, 0.25 mL Evans blue dye 1% (Sigma-Aldrich, Merck Company) was injected into the same locations as the contrast agent. The gentle massage was done on the injected site for 1–2 min. The dissection was performed on the operating table. Colored lymphatic vessels were identified and tracked to the sentinel lymph node.

The excised lymph nodes and mammary tissues were preserved in 10% formalin and submitted for histopathological analysis. The lymph nodes were examined at multiple levels of the paraffin block to optimize metastasis detection. Hematoxylin and eosin staining was used. Histopathological analysis was performed by a histopathologist with expertise in mammary gland pathology.

The sentinel lymph nodes were classified unaffected (absence of metastatic infiltration) and metastatic/affected (presence of metastasis or micrometastasis). When only inflammatory changes were noted, they were interpreted as benign and lymph nodes were classified as unaffected.

### 2.5. Statistical Analysis 

Statistical analysis was performed using IBM SPSS Statistics, software version 20.0, and Microsoft Office Excel 2016. Standard descriptive statistics were used for analysis, means and standard deviations were defined for numeric results. The normal distribution of continuous variables was assessed using the Kolmogorov-Smirnov test. Qualitative variables were compared with histopathological examination using the Chi-Square test. ROC analysis was performed for the evaluation of sensitivity, specificity, positive predictive value, negative predictive value, and overall accuracy of the four techniques defined parameters to differentiate benign from malignant lymph nodes by calculating the area under the curve (AUC). Cut-off values were determined considering the highest value of the Youden index. Significance was defined at a *p*-value lower than 0.05.

## 3. Results

A total of 96 sentinel lymph nodes were evaluated (79 superficial inguinal and 17 axillary lymph nodes belonging to the 71 female dogs enrolled in the study, aged 8–16 years (mean 10.6 ± 1.8). 

The encountered histological type of mammary carcinomas (according to Goldschmidt et al., 2011) [50] is presented in Table 2. The affected mammary gland of each subject and their sentinel lymph nodes, alongside the demographic characteristics, are presented in Supplementary file 1.

Of the total of 96 SLNs evaluated at the histopathological examination, 42 (43.75%) SLNs were classified as benign/unaffected and 54 (56.25%) SLNs were classified as metastatic/affected. The rate of metastasis was 56.33% (40 subjects out of 71).

The sonographic quantitative and qualitative characteristics of the SLNs are detailed in Table 3.

### 3.1. B-Mode Ultrasonography

**Figure 1:**
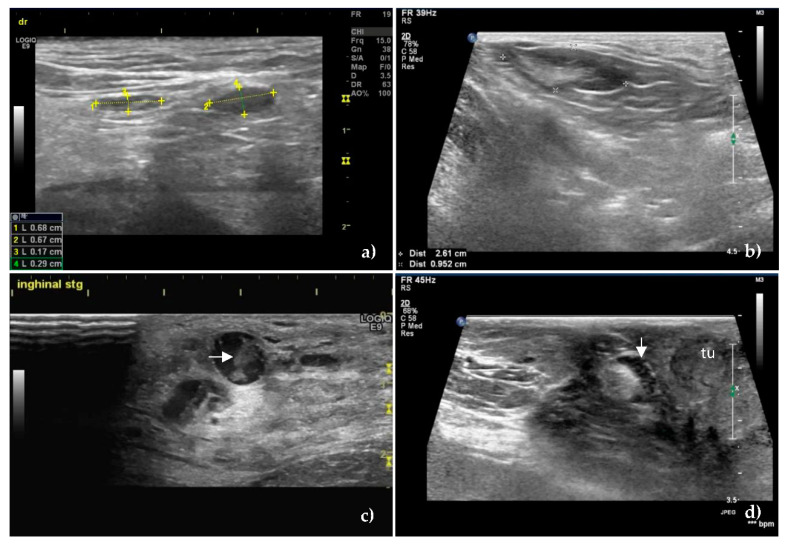
B-mode ultrasound images of unaffected (**a**,**b**) and metastatic, (**c**,**d**) sentinel lymph nodes. Oval shape, S/L ratio less than 0.5, homogeneous echostructure and hyperechoic hilum of unaffected superficial inguinal sentinel lymph nodes in (**a**) and axillary sentinel lymph node in (**b**). The numbers in the image and the lower left corner of Figure 1a represent the long axis (1 and 2) and short axis (3 and 4) measurements of the examined SLNs. The distance between the two “+”signs in Figure 1b represents the measurement of the long axis, and the distance between the two “*×*” signs represents the measurement of the short axis of SLN. The values are found in the lower left corner of Figure 1b. (**c**) Metastatic superficial inguinal sentinel lymph node showing rounded shape, hypoechoic pattern, and inhomogeneous echostructure with coagulation necrosis inside of lymph node (horizontal arrow) as an echogenic structure which leaves no shadows. (**d**) Cortical thickening of a metastatic superficial inguinal sentinel lymph node (down arrow) located near a tumor—tu.

At greyscale US, the S/L axis ratio value >0.55 was the most significant predictive sign for the presence of metastases in the SLNs (Figure 1), having a sensitivity of 83.3% and a specificity of 78.6%.

The mean S/L ratio was lower in the unaffected SLNs (0.50 ± 0.09), compared to the metastatic ones (0.65 ± 0.13) with *p* < 0.001. The same characteristic was maintained in terms of the mean value of the short axis—0.67 ± 0.26 in unaffected SLNs, versus 1.17 ± 0.53 in metastatic SLNs, respectively 1.33 ± 0.46 mean value of the long axis in unaffected SLNs versus 1.83 ± 0.79 in metastatic SLNs.

The homogeneity in the unaffected SLNs was obvious (Figure 1a,b), compared to the inhomogeneous metastatic lymph nodes, (*p* < 0.001) (Figure 1c,d). There was no statistically significant difference between the echogenicity of the two categories of SLNs. The majority of the unaffected SLNs were hypoechoic (66.6%) or isoechoic (23.8%), features which were found in the metastatic SLNs too, rated as hypoechoic (68.51%) or isoechoic (25.92%), *p* = 0.81. The hyperechoic pattern was found in 9.52% of the unaffected SLNs and 5.55% of the metastatic SLNs.

Hilum was visualized in 71.48% of unaffected SLNs and most of the metastatic SLNs—53.70%. There was no significant difference between the two categories, *p* = 0.07, related to the visualization of the hilum.

Regarding the delimitation given by the lymph nodes capsule, no statistical difference was noted between the two categories of SLNs, *p* = 0.057, both unaffected (88.09%) and metastatic lymph nodes (72.22%) having a well-defined capsule.

However, a difference was noted regarding the appearance of the margins in the sense that the metastatic infiltrated SLNs had irregular and blurred margins compared to the smooth, sharp appearance in unaffected SLNs.

### 3.2. Color Doppler Ultrasonography

The Doppler technique certified the presence of a vascular signal in 40 unaffected SLNs and 53 metastatic SLNs, with different pattern between the groups, *p* < 0.001.

**Figure 2:**
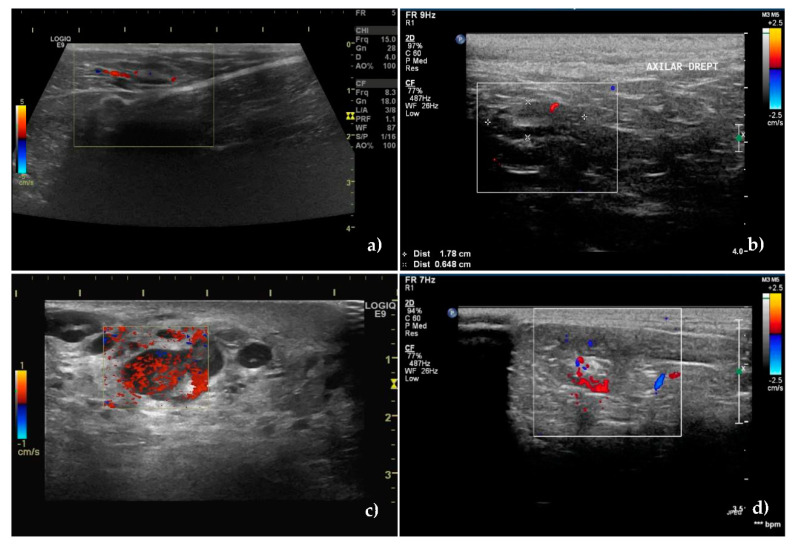
Vessels location and distribution assessed by Color Doppler ultrasound. (**a**) Central, hilar vessels of unaffected superficial inguinal sentinel lymph node and (**b**) unaffected axillary sentinel lymph node. (**c**) Presence of neovascularisation with an abnormal, hilar, and peripheral distribution of vessels in a metastatic superficial inguinal sentinel lymph node. (**d**) Mixed hilar and peripheral pattern with the parenchymal subcapsular location of vessels in a metastatic axillary sentinel lymph node.

In 85.71% of non-metastatic SLNs, blood vessels started from the hilum and were orderly distributed toward the capsule (Figure 2a,b), whereas in 88.88% of the metastatic SLNs, the vascularization had a disordered, chaotic appearance, with predominantly peripherally location (55.55%) or mixed (20.37%) (Figure 2c,d). 12 metastatic SLNs had a perfused hilar region.

Regarding the intranodal vascular resistance, the mean values of the resistivity index (RI), were lower in the unaffected nodes: 0.50 ± 0.14 vs. 0.70 ± 0.13 in the metastatic SLNs (*p* < 0.001).

The same trend was also recorded for the pulsatility index (PI). Its mean value was lower in the unaffected nodes 0.98 ± 0.22 compared to 1.24 ± 0.25 in the metastatic ones.

**Figure 3:**
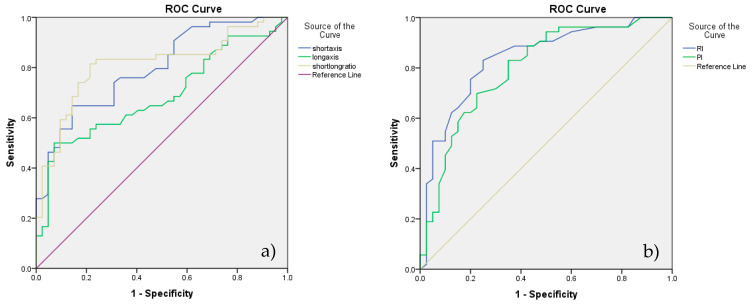
ROC analysis assessing the diagnostic accuracy of B-mode parameters—short axis, long axis, and short/long ratio in (**a**) and Doppler parameters—resistivity index (RI) and pulsatility index (PI) in (**b**).

According to the ROC analysis, the cutoff value obtained for lymph node differentiation, for RI was 0.56 and for PI was 1.02 with 83% SE and 75% SP for RI and 83% SE respectively a 65% SP for PI (Figure 3b).

The accuracy of the two methods was 80% for RI and 75.14% for PI. 

Considering above mentioned analyses, S/L ratio and RI were the best discriminators of the two categories of lymph nodes (Figure 3a,b).

### 3.3. Contrast-Enhanced Ultrasonography

**Figure 4:**
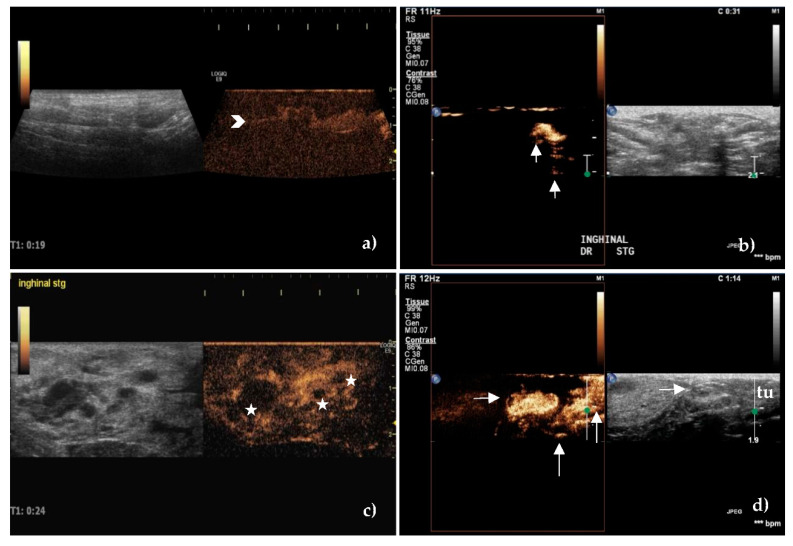
Contrast-enhanced ultrasound of sentinel lymph nodes after peritumoral administration of contrast agent. (**a**) Complete, homogeneous enhancement of an unaffected axillary sentinel lymph node with an evident hyperechoic lymphatic vessel—chevron arrow. (**b**) Intense enhancement of an unaffected superficial inguinal sentinel lymph node. Note the lymphatic vessels that bypass the lymph node leading to the next lymph node station—vertical arrows. (**c**) Inhomogeneous enhancement of a metastatic superficial inguinal sentinel lymph node. Areas with enhancement defects are present—stars. (**d**) The focal cortical area with no enhancement—horizontal arrow, of a metastatic superficial inguinal sentinel lymph node located in the vicinity of a tumor (tu) of the inguinal mammary gland. Note the multiple afferent lymphatic channels as hyperechoic linear structures that approach the lymph node and the contrast agent that was injected peritumorally—vertical arrows.

After peritumoral injection of SonoVue, the lymphatic vessels were identified as hyperechoic and well-defined linear structures Initially, the route of the lymphatic vessels was superficial, then became deeper close to the SLNs (Figure 4).

Three types of lymph node enhancement have been established: 1. intense or moderate but homogeneous enhancement without areas with no contrast; 2. inhomogeneous or partially inhomogeneous with the presence of areas without contrast, either peripherally or centrally, and 3. no enhancement.

The enhancement patterns were quantified as intense, centripetal, homogeneous, without enhancement defects in most unaffected SLNs (76.19%), belonging to type 1, (Figure 4a,b) compared to the inhomogeneous pattern (81.48%), with the presence of areas with no contrast in the metastatic ones (*p* < 0.001), belonging to type 2 (Figure 4c,d).

There was no enhancement in 4 female dogs (4 lymph nodes belonging to the axillary lymph center and 2 lymph nodes belonging to the superficial inguinal lymph center) belonging to type 3.

Considering that type 1 is found in unaffected and type 2 and 3 in metastatic SLNs, we obtained a sensitivity of 88.89%, a specificity of 76.19% with PPV 82.76%, NPV 84.21%, and good accuracy of 83.33%.

Regarding the contrast transit times, the mean values of the wash-in time of 18.12 ± 7.15 (s) in unaffected SLNs was not statistically different from the metastatic ones, 15.34 ± 7.23 (*p* = 0.07), having 64% SE and low SP of 59%, with PPV 68.15%, NPV 57.07%, and 62.92% accuracy. The established cutoff value was 17.5 s.

The shorter and more marked wash-out time of 104.26 ± 39.67 (s) in the metastatic SLNs, compared to 151.48 ± 34.62, in unaffected SLNs, (*p* < 0.001), was a better predictor of metastatic infiltration. If a cutoff value of 133 s for wash-out time determined by ROC analysis was considered, the SE and SP were 84% and 74.36%, respectively, with PPV 80.81% and NPV of 78.33% and 79.78% accuracy.

Enhancement patterns and contrast transit times are presented in Table 3.

### 3.4. Real-Time Elastography

Using the score proposed by Alam et al. [49], which quantified the relative proportion of the areas with low deformability (or high rigidity), a cutoff value between scores 2 and 3 was established.

**Figure 5:**
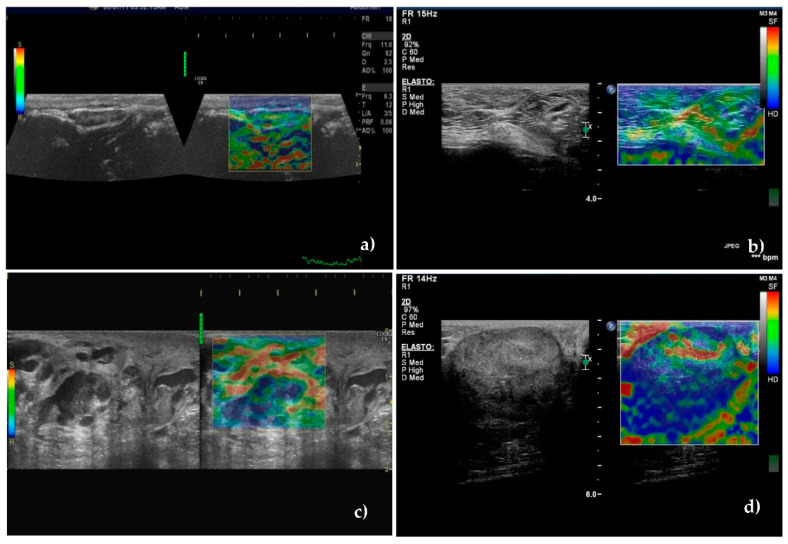
Sonoelastographic images of unaffected—(**a**,**b**)—and metastatic—(**c**,**d**)—sentinel lymph nodes. (**a**) Unaffected axillary sentinel lymph node showing soft appearance—total green with small blue areas corresponding to score of 1. (**b**) Moderately soft superficial inguinal sentinel lymph node with small scattered blue areas corresponding to score of 2. (**c**) Most of the superficial inguinal lymph node is blue with small green areas inside suggesting necrosis, corresponding to score of 4. (**d**) Hard-blue area occupying entire lymph node, with green rim corresponding to score of 5.

Among metastatic SLNs, 88.9% were classified in scores 3, 4, or 5 (hard). These scores were recorded in only 9.5% of the unaffected SLNs. Scores 1 and 2 were recorded in 90.5% of unaffected SLNs (Figure 5a,b) and in 11.1% of metastatic SLNs, (*p* < 0.001) (Figure 5c,d).

The classification of SLNs in a certain score is presented in Table 4. Stiffness was significantly higher in metastatic SLNs compared to the unaffected ones (Figure 5) having a very good SE, SP, PPV, NPV, and accuracy to identify the metastatic lymph nodes, of 88.9%, 90.5%, 89.69%, 86.36%, and 89.71%.

**Figure 6:**
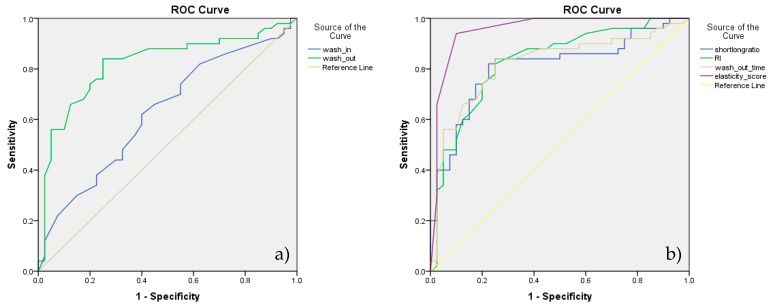
(**a**) ROC analysis assessing the diagnostic accuracy of contrast-enhanced ultrasound (CEUS) parameters, wash-in time and wash-out time; (**b**) ROC analysis assessing the diagnostic accuracy of significant parameters of the four techniques—B-mode, Doppler, CEUS, and elastography.

Considering the above mentioned analysis of CEUS, wash-out time was a better discriminator of the two categories of SLNs—Figure 6a. ROC analysis assessing the diagnostic accuracy of significant parameters of B-mode, Doppler technique, CEUS and real-time elastography is presented in Figure 6b.

The diagnostic performance of grayscale US, color Doppler, CEUS, and elastography is summarized in Table 5.

According to our results, the highest diagnostic accuracy for prediction SLNs metastases was given by the elasticity score (89.71%) followed by the S/L ratio (81.25%) and RI (80.0). The statistical significance of the AUC was preestablished according to the following qualification: 0.500–0.600—improper, failure; 0.600–0.700—poor, weak; 0.700–0.800—fair; 0.800–0.900—good; 0.900–1—excellent.

Combining the most significant parameters, namely short/long ratio, resistivity index, wash-out time and elasticity score, we obtained the best diagnostic performance with 92.27% accuracy, 94% SE and 90% SP.

Considering previous reports [48,51] and based on our results obtained by ultrasonographic examination and their correlation with the histopathological examination, we developed a US examination algorithm using the significant parameters of the four techniques—grayscale US, color Doppler, CEUS, and elastography. Each parameter receives a 0 or 1 score depending on its significance. Thus, the classification of SLNs in one of the two categories, unaffected or metastatic, was appreciated. The data is presented in Table 6.

### 3.5. Lymphatic Mapping

After peritumoral injection of Evans Blue Dye, the identification rate of SLNs was 98.5% (70/71 subjects) average of 1,3 SLN/subject.

Out of 70 subjects, in 46 (65.71%) a single SLN was identified and in 24 subjects (34.28%) mammary tumor drainage was performed by 2 SLNs. In 8 subjects (11.28%) presenting A1, A2, and I tumors, the drainage was made both cranially (by proper axillary lymph node—5 subjects- or accessory axillary lymph node—3 subjects) and caudally (by the superficial inguinal lymph nodes)—in all subjects. In 2 subjects with T1 mammary gland tumor, a lymphatic vessel was identified which branched off, giving off an obvious lymphatic vessel which drained into the proper axillary lymph node and another lymphatic vessel bypassing the proper axillary lymph node to drain into the cranial sternal lymph node. The cranial sternal lymph node was considered also SLN.

The drainage pattern of each subject is presented in Supplementary file 1.

## 4. Discussion

The clinical applicability of US is fully recognized in the primary assessment of superficial drainage within a well-defined territory. Based on the hypothesis that the sum of available US methods can differentiate between unaffected and metastatic SLNs, our research analyzes and describes the normal and pathological aspects of draining lymph nodes of mammary carcinomas in female dogs.

In current practice, the surgical therapeutic approach by local mastectomy or excision of the whole unilateral mammary chain is accompanied by ipsilateral lymphadenectomy, if SLNs are identified intraoperatively [52,53,54]. As in breast cancer in women, the appearance of metastases in the lymph nodes correctly stages cancer, guides treatment, prognosis, and evolution [2,3,4,53,54,55]. There is debate about the influence of the lymph nodes metastatic burden on the histologic malignancy grade, prognosis, and disease-free survival time of both women and bitches [3,54]. Micrometastases (MICs) and isolated tumor cells (ITCs) detected by immunohistochemistry (IHC) are the subject of recent studies [4,55], but their influence on cancer evolution has not yet been clearly established, but there is a chance that in the absence of IHC these MIC and ITCs will not be detected [55].

Although it has been stated that there are no differences between the evolution of cases with negative lymph nodes and those in which the presence of MIC or ITCs was determined by IHC [3], future studies are needed to evaluate lymph nodes from this point of view. At the same time, the existence of correlations between the presence of MIC and ITCs and the ultrasonographic patterns must be appreciated. Excision of sentinel lymph nodes draining the tumoral mammary glands, without a prior evaluation, is unjustified from two perspectives. The first reason is that an SLN must be certified as a certain drainer of the tumor. Secondly, the excision of a benign SLN deprives a relatively large territory of lymphatic drainage, which also drains other structures, leading to the well-known secondary effects. These effects are encountered in both humans and animals: limb lymphedema, pain, diminished peripheral nerve sensitivity and reducing the local defense capacity [13,55,56]. That is why, through this study, we tried to avoid inguinal or axillary clearance and we created an evaluation algorithm using the simplest and most available ultrasound methods.

### 4.1. B-Mode Ultrasonography

It has been shown that on the grayscale US the S/L ratio is an important criterion for differentiating the categories of SLNs in both humans and animals [18,28,41,57,58].

The higher observed ratio in the malignant SLNs is due to the tumoral infiltration that causes a change in shape (the lymph node tends to be rounded), effect quantified by calculating this ratio [57,59]. Likewise, the local and regional inflammatory processes cause a clear rounding of the lymph nodes that drains the affected territory [30]. Compared to the metastatic lymph nodes, (whose modification is most often unilateral), depending on the path of the related lymphatic pathway that carries the malignant cells, enlargement of inflammatory lymph nodes, is uniform.

A similar feature regarding the uniformly-rounded shape of malignant lymph nodes is found in lymphomas, frequently diagnosed in dogs [17,18,28,58,60,61]. Most animal studies report statistically significant S/L ratio values, without specifying SE and SP values in discriminating lymph node categories.

In our study, the cutoff value of the S/L ratio obtained on the ROC analysis was 0.55 having 83.3% SE and 78.6% SP, these values being found in other reports too. Cited sources reported a SE of 48.8–87.1, 86.7, 86.8, 93% [28,48,62,63] and 55.6–97.3, 67.2, 72.5, 53% SP. In our study, the S/L ratio presented a good accuracy of 81.25% for the identification of metastatic SLNs.

In a recent study, Silva et al., (2018), [30] showed that the S/L ratio has a moderate discriminative power of around 60% in differentiating the altered lymph nodes. Mean values of this ratio calculated in free, altered, and metastatic lymph nodes, were non-specific and lower in comparison with other studies, namely 0.38, 0.37, and 0.34 for axillary lymph nodes and 0.47, 0.49 and 0.53 for superficial inguinal lymph nodes. The mean values of the S/L ratio in our study, 0.50 for unaffected SLNs and 0.65 for metastatic SLNs, were higher compared with the above-mentioned study.

Considering the clinical anatomy, there are significant differences between the shape of the normal lymph nodes depending on the topography and the lymphatic drainage basin which they serve, e.g., mesenteric lymph nodes in dogs are more elongated than medial iliac lymph nodes; superficial cervical lymph nodes and medial retropharyngeal lymph nodes have a much more fusiform appearance compared to the proper axillary lymph nodes or submandibular lymph nodes; benign parotid lymph nodes are more rounded than superficial cervical lymph nodes; the shape of popliteal lymph nodes is generally rounded [17,41,42,58,59,64,65]. Under these conditions, the S/L ratio must be combined with other techniques for a certain SLN classification.

The size of the lymph nodes established by measuring the two axes is not a relevant criterion for the differentiation of the metastatic lymph nodes. In most studies, the measurements of the two axes are related to the ratio calculation. Standard assessments cannot be made, either on deep lymph nodes or superficial lymph nodes, as long as the size of the lymph nodes is bodyweight, breed, body mass index, age or sagittal and longitudinal thoracic diameters- dependent [59,64,65] and the presence or absence of associated pathology. Certain studies report the average values established for the submandibular, medial retropharyngeal, cervical superficial, proper axillary, and superficial inguinal, normal lymph nodes, ranging from 1.35, 1.9, 1.48, 1.57, 1.79 cm for the longitudinal axis, 1.00, 0.98, 0.85, 0.81, 0.68 cm for the transverse axis and 0.41, 0.55, 0.41, 0.65, 0.31 cm for the sagittal axis [59,64,65].

In pathological cases, these values increase significantly, reaching up to 2.61–5.5 cm for the longitudinal axis and 1.15–2.8 cm for the transverse axis [17,28] being comparable to the average values recorded in our study, respectively 1.33 cm for LA and 0.67 cm for SA in unaffected SLNs and 1.83 cm for LA and 1.17 cm for SA in metastatic SLNs.

The fact that SA had better accuracy (73.96%) compared to LA (68.75%) has an anatomical explanation. First of all, normal afferent or potential vessels that carry the malignant cells, approach the node on the capsular level, causing SA growth in the first phase. Secondly, the arrangement of the superficial lymph nodes is, either in the subcutaneous cellular tissue or in the intermuscular spaces with the long axis oriented towards the lymphatic drainage, which relatively limits the expansion of LA.

Another aspect related to the variability of lymph node size is age-dependent, with an inverse correlation between age and size [66,67,68]. This is due to the immunosenescence and atrophy of the lymph nodes based on the specific degenerative changes responsible for the altered immune response and an increased rate of cancers in old ages.

Under these conditions, the shape and size of the lymph nodes cannot be used as a unique criterion for differentiation. In contrast, their size may provide important data for monitoring treatment response, especially in lymphoma, by significantly decreasing the LA value [69].

In our study, the SLNs margins or capsule was not a significant criterion for differentiating the two categories, similar to other reports [17,30,42] in animals, but different from reports of sentinel lymph nodes margins in humans [48,70]. The majority of both unaffected (88/09%) and metastatic (72.22%) SLNs in our study, were well delimited to surrounding tissue. The sharp appearance of most of the capsules in the metastatic SLNs, is due to capsule and nodal parenchyma infiltration with tumor cells (that causes an increase in acoustic impedance difference between the inside and the surrounding tissue [42,71,72]).

The reports regarding the appearance of the lymph node capsule are very heterogeneous. Some stated that both, benign lymph nodes and malignant ones have irregular contour but well-defined margins [28] but most of the malignant nodes were diagnosed with lymphoma and a small number were represented by metastasis of local tumors. On the other hand, studying cervical lymph nodes in healthy dogs, it has been shown that benign lymph nodes can have both irregular and smooth margins [65].

The internal structural features of the SLNs examined in our study are consistent with studies that evaluated superficial or profound normal or pathological lymph nodes in animals and humans. It has been proved that homogeneity is a specific feature of benign lymph nodes, compared to the inhomogeneous appearance of malignant lymph nodes [18,41,48,73,74]. Focal cortical nodules, intranodal necrosis, calcifications are responsible for the inhomogeneous appearance of the malignant lymph nodes [41]. These structural changes are obvious in superficial SLNs, compared to the deep ones, where these changes may be influenced by certain artifacts or by the difficulty of the examination.

In contrast, other studies have reported that these two characteristics (homogeneity and inhomogeneity) are not always associated with the presence of metastases in lymph nodes. Cited sources state that the categories of sentinel lymph nodes cannot be differentiated considering only this criterion [30] as long as the lymphomatous nodes are homogeneous similar to the benign ones [17,42]. These studies differ from the present one, both in the variety of lymph nodes evaluated (submandibular, superficial cervical, popliteal, medial iliac, mesenteric, hepatic, or even cranial sternal and cranial mediastinal) as well as the variety of disorders that have determined the lymph node structural changes.

The lack of significant difference in the echogenicity of the malignant lymph nodes compared to the unaffected sentinel lymph nodes from our study is similar to other reports regarding this criterion [17,28,65]. Although in humans, the hypoechoic pattern of the lymph nodes is commonly associated with malignancy [41,48,72], in animals, most of the lymph nodes have hypoechoic or isoechoic appearance, while the hyperechoic pattern is found in both malignant and benign lymph nodes.

In our study, the hypoechoic pattern was more prevalent compared to isoechoic or hyperechoic appearance. It should be specified that the echogenicity of a lymph node is not necessarily related to its homogeneity as long as the heterogeneous lymph nodes can be cataloged in some circumstances as hypoechoic [18,19]. Furthermore, the lymph node cortex that contains few sinuses in a connective tissue network is responsible for the hypoechoic pattern of the lymph nodes, because the interfaces are few and the reflection is poor. A hypoechoic cortex (>3 mm in size) is considered to have metastatic infiltration in both animals and humans [54,75]. However, attention must be paid to this criterion because for the measurement it is necessary the presence of a hilum and on the other hand in animals, the size of the lymph nodes is dependent on breed and condition. Therefore, malignant lymph nodes may appear hypoechoic and homogeneous or heterogeneous/inhomogeneous, as cited for some mammary tumors, the heterogeneity being produced by the clusters of malignant cells on conjunctive support [43,44,50,52]. Intranodal necrosis is generally heterogeneous, depending on the stage of necrosis, being seen as coagulation or liquefaction necrosis [41,71].

On the other hand, the echogenicity is debatable, as long as different locations of the lymph nodes induce different appreciations. For example, the medial retropharyngeal lymph node (located caudally to the digastric muscle, ventrally to the long muscle of the neck, covered by the sternocephalic muscle and the mastoid end of the brachiocephalic muscle) may appear hyperechoic in relation to the above-mentioned muscles or isoechoic to the deeper adjacent portion of the salivary gland [65]. The proper axillary lymph nodes examined after pulling the anterior limb extended forward may appear hyperechoic if we refer to the large round muscle insertion, or slightly hypoechoic if we refer to the subscapular and axillary veins [20]. The accessories axillary lymph nodes are hyperechoic if we compare them to the dorsal edge of the ascending pectoral muscle. The most important conclusion from the comparisons of the echogenicity is that this cannot be considered a reliable parameter for differentiation of the metastatic from the benign or unaffected lymph nodes.

The hilum was visualized in the majority of SLNs, both unaffected (71.48%) and metastatic (53.70%), as previously has been reported in animals [17,19,28]. Not the same conclusions were recorded in certain studies related to the SLNs that drain the female breast cancer [70,75,76] or in different neoplasms in animals [18,42,51] that associated the presence of metastases with the lack of hilum echogenicity. The metastatic infiltration of the hilum occurs in the relatively late stages of metastatic dissemination, in which the echogenic structures represented by multiple medullary sinuses, are replaced by the tumor cells, reducing the reflectivity of the interfaces [41,71,77]. The presence of a thin, effaced hilum associated with cortical focal hypoechogenicity raises the suspicion of metastatic infiltration [51]. It is also considered that ischemic degeneration at the level of the hilum may be a consequence of its visualization as a thin, hyperechoic structure [19,78] an aspect also encountered in this study. In these conditions, on the grayscale US, the presence or absence of hilum is not reliable enough in differentiating between metastatic and benign lymph nodes.

### 4.2. Color Doppler Ultrasonography

The evaluation of lymph node vascularization by the Doppler technique offers a very good appreciation of their status, many authors suggesting the possibility of differentiation the metastatic from the benign or unaffected lymph nodes, both in humans and animals [41,42,51,58,71,79]. It has been stated that with respect to the histopathological examination, Doppler US is capable to detect and evaluate with great accuracy the blood flow in superficial lymph nodes [58].

In the present study, we defined the presence of the Doppler signal in three locations: hilar, peripheral, and mixed. The absence of the vascular signal was recorded in 3 SLNs. The absence of the Doppler signal can be recorded in the benign or unaffected lymph nodes of small size. In these nodes, the small vessels contain a small number of red blood cells at one time, which leads to the decrease of the Doppler signal intensity. A similar situation is noted in the metastatic lymph nodes in which the necrosis areas are lacking Doppler signal [71,80,81]. Most unaffected SLNs in the present study showed hilar vascularization (85%), the anatomical place of entry of the lymphatic vessels, but hilar and mixed vascularization was found in the metastatic lymph nodes too. A similar pattern was found in SLNs that drain the breast cancer in women [70,76,82].

The presence of hilar vascularization in metastatic lymph nodes is found in the early stages of metastasis, where the hilar vessels are not yet destroyed by the tumor invasion. The peripheral identification of the Doppler signal in most of the metastatic sentinel lymph nodes in our study is similar to other reports that concluded that this aspect is associated with the presence of metastases in lymph nodes having high specificity and variable sensitivity [19,58,77,83].

Contrary to these claims, evaluating the vascularization of malignant and benign tumors of the mammary glands in bitches, Soler and collaborators (2016) [43] showed that the peripheral distribution of vessels detected by the Doppler technique is significantly different in benign tumors compared to malignant ones. They concluded that this is because initially benign tumors have wider vessels at the periphery of the tumor while malignant tumors in need of increased vascular support show the mixed type of vascular distribution [43]. Tumor cells that first approach the lymph node in the cortical sinuses and then medullary sinuses, by secretion of angiogenic factors, determine angiogenesis and recruitment of peripheral vessels making peripheral identification possible through the Doppler technique [19,41,71,81].

In our study in 88% of the metastatic SLNs, the distribution of vessels had a disordered, chaotic distribution, this pattern being previously reported [48,84]. This feature is determined by the shape changes caused by the invasion of the tumor cells that displace the ordered pathway of the vessels from the hilum to the lymph node parenchyma and, on the other hand, it is the result of the development of new blood vessels induced by the tumor angiogenic factors which determine anarchic and distorted growth of newly formed arterial vessels [81,83,84,85,86].

The analysis of vascular indices revealed their high values in the metastatic lymph nodes, the predictive cutoff values established on the ROC analysis being 0.56 for RI and 1.02 for PI. The increase of these values in the metastatic lymph nodes is a consequence of the compression given by the tumor cells to the parenchyma and the blood vessels causing an increased vascular resistance but also the induction of desmoplasia (fibrous tissue hyperplasia). These events multiply the structural changes leading to a greater increase in the vascular resistance [71,87]. The cutoff values, for RI and PI for which we obtained the best sensitivity and specificity, are lower compared to other values for dogs, namely 0.67 and 1.02 [87] 0.68 and 1.49 [42] 0.69 and 1.49 [28] or in humans 0.7 and 1.4 [71]. The differences are probably because these studies evaluated different lymph nodes, located both superficially and deeply, being considered SLNs in different neoplasms, compared to the uniform group of this study in which the axillary SLNs and superficial inguinal SLNs were evaluated.

To the best of our knowledge, there is no report regarding the vascular indices of SLNs that drained strictly the neoplastic mammary glands in female dogs. In another research regarding the status of draining lymph nodes of neoplastic mammary glands in bitches [30], in addition to grayscale and ARFI Elastography, Doppler examination evaluated only the presence of the vascular signal and its location without determining the RI and PI. Bellota et al. (2019) [28] besides the superficial cervical, submandibular, and popliteal lymph nodes that drained the locoregional tumors, investigated only 1 axillary lymph node and 12 superficial inguinal lymph nodes that drained mammary carcinomas. In our study, we found a good accuracy of 80% for RI in distinguishing between the two categories of lymph nodes.

### 4.3. Contrast-Enhanced Ultrasonography

Considering that the high density of peritumoral lymphatic vessels is a reliable criterion for predicting the presence of metastases in SLNs [5,85,88] and taking into account that SLN may be other than locoregional lymph nodes [33,34], we injected the contrast agent (SonoVue), peritumorally. In a preliminary study of our team, we administered the contrast agent both intravenously and peritumorally, focusing on the description of the aspects encountered [26]. The cases were few and no relevant statistics were performed.

The peritumoral administration of SonoVue in the present study was considered optimal for the identification of the true lymph node draining the carcinoma in question. In this way, we tried to avoid examining another regional lymph node that does not drain the carcinoma. In this respect, Goldberg and collaborators (2004; 2005) [89,90] showed that by modifying the injection site of AC with only 1 cm, different lymph nodes could be identified as SLNs than those of the tumor in question. On the other hand, the functional anatomy of the region must be considered. Intradermal or subareolar administration of a dye or radioactive tracer certainly identifies a large number of lymphatic vessels, but for the contrast agent to penetrate the lymphatic vessels, higher interstitial pressure than that of the dermis is required [91,92].

We considered that subcutaneous peritumoral administration of CA is justified due to the increased interstitial pressure given by the tumor itself and the tumor-induced lymphangiogenesis which causes an increase in peritumoral lymphatic density. From the injection site, the lymphatic vessels were identified in all subjects as hyperechoic linear structures that led to corresponding SLN. Similar reports were made after intradermal of SonoVue in pigs and women [93,94,95] or subdermal injection in the dog [10]. The dose of contrast agent that we have used for the peritumoral injection was determined based on previous research of our team [26] and other reports [46,47]. The doses used by reference studies are variable and there is still no protocol regarding this aspect. The learning period of the procedure is relatively short and does not require a large number of cases, and can be successfully performed in the clinic.

In the present study, in six subjects no SLN was enhanced. The explanation could be related to the obstruction of the associated lymphatic vessels by the tumor thrombi or the reduced size of the unaffected non-enhanced SLNs.

In our research we defined 3 types of enhancements: 1. homogeneous, complete, without enhancement defects; 2. inhomogeneous, incomplete, with areas without CA and 3. difficult to quantify/no enhancement as it was previously reported [47,96]. Type 1 was associated with benignity and type 2 and 3 were associated with metastatic infiltration. Other authors [48,95] have defined 4 types of enhancements: 1. uniform, complete; 2. uneven with the presence of areas with high or low enhancement; 3. peripheral complete or incomplete ring enhancement with low or no enhanced areas inside; 4. no enhancement associated with a related lymphatic vessel. Type 1 were considered negative nodes and types 2, 3, and 4 were considered metastatic. In our study, the majority of metastatic SLNs had type 2 enhancement (44 SLNs out of 54 metastatic SLNs), but the inhomogeneous aspect of enhancement was also found in 8 SLNs that were diagnosed as negative on histopathological examination. This may occur due to hyperplasia of the lymphoid follicles, adipose tissue deposits, chronic inflammation, or proliferation of fibrous tissue, leading to the uneven and inhomogeneous distribution of the contrast agent [95,96].

The sensitivity and specificity of 88.89% respectively 76.19%, with PPV of 82.76% and NPV of 84.21% obtained regarding enhancement type, are comparable with another reports [47], in which a SE of 81.8% and SP of 86.2% with PPV 75.0% and NPV of 90.3% was obtained. These results are different from those obtained by Liu and collaborators., (2019), [95] which obtained a high SE of 98.04% and a low SP of 49.23%. According to our results, after the peritumoral injection of the contrast agent and the enhancement type analysis, we obtained a good accuracy of 83.33% in differentiating the categories of SLNs.

Perfusion times analysis returned surprising results in the sense that wash-in time was not different in metastatic vs. unaffected SLNs, (*p* = 0.07). Studies performed in women after IV administration of CA and quantification of perfusion times are very heterogeneous, reporting different SE, SP, PPV and NPV of 59%, 87%, 63%, and 85%, [97] or 92.6%, 76.0%, 80.6%, and 90.5% [98]. Although it was specified that in metastatic SLNs, wash-in time is shorter (<15.90 s) [99] and clear, we did not observe this aspect. Indeed, overall, the wash-in time was faster and shorter in the metastatic SLNs, but there was no statistical difference between the mean values of the two categories (17.35 s in unaffected SLNs vs. 15.12 s in metastatic SLNs).

In contrast, wash-out time was significantly different between unaffected and metastatic SLNs (149.48 s in unaffected SLNs vs. 104.12 s in metastatic SLNs) but our values were higher than other reports, the cutoff value being <133 s compared to <60 s previously established [100] for metastasis. Regarding these results, it should be taken into account that CA was peritumorally injected, under these conditions the longer duration of the perfusion times is explained because migration through the lymphatic pathways lasts longer than the intravenous route. In metastatic SLNs washout time was shorter than in unaffected SLNs, *p* < 0.001, having 84% SE, 74.4% SP, PPV 80.81%, NPV 78.33%, and 79.78% accuracy. However, we consider that these results should be taken into consideration with caution, as large-scale studies are needed to validate this method.

The centripetal enhancement revealed in our study in both unaffected and metastatic lymph nodes is because CA has entered lymph nodes on the related afferent lymphatic pathways, approaching the lymph node at the capsule level, following anatomical pathways. Comparatively, after IV administration, a criterion of differentiation is the centrifugal enhancement in unaffected SLNs and centripetal one in the metastatic SLNs [98,101]. The appearance is of a disorganized type, with areas of unequal vascularization, achieved through several vascular pedicles whose presence is explained by subcapsular neoangiogenesis induced by subcapsular cortical metastases [102]. Regarding the enhancement direction, comparative studies are needed to establish if the administration of the contrast agent should be done both IV and peritumoral.

### 4.4. Real Time Elastography

Another current ultrasonographic technique applied in our research, real-time elastography, had the highest accuracy—89.71% in SLNs differentiation—obtaining 88.9% SE, 90.5% SP, PPV—89.69%—and NPV—86.36%. The high elasticity score of the metastatic SLNs between the values 2 and 3, of the five-point scoring system, was statistically significant to differentiate between the two categories corroborating other reports [29,103]. It is known that the stiffness of metastatic lymph nodes is much higher compared to adjacent tissue or benign lymph nodes in humans and animals [30,49,104,105] and following the compression exerted by the transducer, their displacement is absent. These findings are correlated with palpable clinical evaluation, in which the metastatic lymph nodes are much harder and well anchored in the adjacent tissue [25].

Elasticity scoring systems are based on color-coded elastograms that allow visual appreciation of the proportion of hard tissue in a lymph node, relative to adjacent structures [29,106,107]. On the other hand, due to its components, the cortical area of a lymph node is slightly harder than the medullary, even in benign lymph nodes [108]. However, metastatic lymph nodes are specifically characterized by increased cortical stiffness [109,110]. This is due to multiple factors, including focal cortical stiffness caused by metastatic cell islands, tumor infiltration associated with desmoplasia, and increased angiogenesis and lymphangiogenesis [107,111,112]. Metastatic lymph nodes with areas of necrosis are less hard than those without necrosis. All these structural changes are qualified in different elasticity scores from four, five, or even eight patterns [49,104,109], each of which establishes cutoff value between scores 2 and 3 or between scores 3 and 4 in an eight-point scoring system. Elasticity scores were initially applied in the evaluation of the superficial cervical lymph nodes, but the same score was adapted to SLNs of mammary glands. Although Seiler and Griffith (2017) [29], using both, a four- and five-point scoring system to discriminate cervical lymphadenopathies, stated that the value of the four-point and the five-point score is similar in differentiating the metastatic from the benign SLNs, we applied the five-point scoring system. We applied Alam’s elasticity scoring system because this score also analyses the possibility of the presence of necrotic areas in metastatic SLNs. Compared to the score of Bhatia [104] and compared to other studies [29,106,109], Alam’s scoring system [49] had the highest accuracy of 89% with 83% SE, 100% SP, PPV of 100% and NPV of 78%. Even if the accuracy of the method is high in the above-mentioned study, studies in the field in animals are quite heterogeneous, obtaining various SE values of 53–67% to 83%, 85% and values of 75% and 80–83%, 100% for SP [28,30,103]. Moreover, there is no standardized elasticity scoring system for SLNs that drains the mammary glands with tumors in bitches, the score applied by us being extrapolated from the human evaluation. However, in our study, the elasticity stiffness scoring system provided the best results in differentiating the lymph node categories. These results are consistent with a recent study in which using Acoustic Radiation Force Impulse (ARFI) elastography to identify metastases in axillary and inguinal lymph nodes in dogs with mammary tumors, it was shown that ARFI shear wave velocity (SWV) identified with excellent accuracy (around 90%), the presence of metastases in SLNs [30]. In a recent study, using qualitative assessment (4-point elasticity scores) and semi-quantitatively (mean hue histogram and stiffness area ratio) of mandibular lymph nodes in dogs, the authors found 100% SE and 92% SP for hue histogram and 86% SE and 100% SP for stiffness area ratio in malignancy prediction [103].

Share wave elastography is a novel technique that performs absolute measurement of stiffness in kilo-pascal (kPa) units. Recently two-dimensional Shear Wave Elastography (2D SWE) was used to evaluate liver, pancreas, kidney, thyroid, prostate, and submandibular, retropharyngeal, axillary and inguinal lymph nodes in nine Beagle dogs. All lymph nodes were visualized with a uniform color map and constant contour lines on 2D-SWE while SWS (share wave speed) was not significantly different between lymph nodes [113]. All these procedures are still in their experimental use in veterinary medicine, with few studies and special requirements in terms of used software. A limitation of the qualitative elastography used in the present study is that this method is examiner-dependant. We applied this method in our algorithm because the elastography module is incorporated in most devices used in current practice. The evaluation and assignment of a score can be easily done by practitioners.

### 4.5. Lymphatic Mapping

SLNs identification after peritumoral injection of the dye provided similar results to other reports that used the dye injections at various locations to identify SLNs in female breast cancer [5,114,115,116]. The most commonly used identification technique is the association of 99mTc (labeled colloids) with a dye (patent blue, isosulfan blue, or indocyanine green) injected after colloid administration, and detection by scintigraphy, SPECT or using a gamma probe [8,72,117,118,119,120].

In recent years, multiple attempts have been made to improve lymphatic migration by developing new small radiocolloids to which is added either the dye or other molecules. These molecules, by binding to macrophage receptors and dendritic cells in lymph nodes, increase the possibility of retaining the identification agents inside of lymph nodes [116,118,121]. In female breast cancer, the use of the fluorescent spectrum emitted by indocyanine green (ICG) detected intraoperatively was most commonly used, with an increased identification rate of over 95% [118,122]. The newer identification technique using superparamagnetic iron oxide (SPIO) and its ability to be identified due to its magnetic properties using a detector, provided slightly higher results of the identification rate of 97.6% compared to the classical identification using radiocolloid and blue dye—96.8% [123,124]. Tracer injection is usually peritumoral or subareolar. Even today, there are debates about the injection site, and whether the same SLNs are identified after administration in different locations.

We administered both the contrast agent and the dye peritumorally because we started from the hypothesis of lymphatic drainage variability in bitch demonstrated by numerous studies [13,33,34]. These studies have shown that mammary glands with tumors drain variably, recognizing as sentinel lymph nodes other lymph nodes that are not known to be characteristic of mammary glands drainage, respectively, cranial sternal lymph nodes, or superficial cervical lymph nodes for cranial mammary glands, and popliteal lymph nodes or medial iliac lymph nodes for the caudal mammary glands. The fact that we encountered in a subject a bypass vessel of the proper axillary lymph node that drained into the cranial sternal lymph node, sustains this statement. On the other hand, different pathways of lymphatic vessels, namely superficial, deep and penetrating lymphatic channels, in both women and bitches are also recognized [33,34,93,114,115,125].

However, at present, it is not possible to specify the route of the lymphatic draining vessels of a mammary gland with a tumor. Basically, a tumor is drained by both the deep and the superficial network. What is known is that peritumoral lymphatic density is increased, either on account of pre-existing vessels or those newly formed by tumor-induced lymphangiogenesis itself [126,127]. Under these conditions, we cannot omit these newly formed vessels that may have a different route than those coming from the lymphatic network of the mammary gland. Lymphatic drainage regardless of a certain territory or organ is uneven and unpredictable [25,35]. We consider that the peritumoral administration of both the dye and the contrast agent is justified to identify the true SLN.

Although this is a relatively large study using four ultrasonographic methods, some limitations can be stated. The first would be related to the small number of evaluated cases; we evaluate the lymph nodes as study units, therefore studies with a higher number of cases are needed. Another limitation could be given by the fact that the images were acquired by the same researcher, but by using two devices in the evaluation we tried to solve this impediment. All procedures were supervised by two experienced practitioners, one in human imaging and one in veterinary imaging. The ultrasonographic methods used in our study were very simple and can be performed by any practitioner who deals with the evaluation and treatment of mammary gland tumors in dogs and does not require a long period of learning. Moreover, the costs associated with performing these techniques are low because current ultrasound devices have incorporated contrast and elastography software. That is why even for the more advanced techniques like CEUS and elastography we used their simplest approaches. Subjectivism can be controlled by combining all the techniques for obtaining a good diagnostic performance. On the other hand, the methods are not fully standardized in terms of evaluation of lymph nodes that drain mammary gland tumors in the females’ dog requiring extensive studies to validate the ultrasonographic algorithm. The differentiation between various pathologies in which sentinel lymph nodes are the first station of metastatic dissemination or independent pathology (lymphoma) should be made. The addition of the quantitative parameters of the CEUS and elastography techniques will lead to a substantial increase in the diagnostic performance of the ultrasonographic evaluation algorithm.

## 5. Conclusions

The accuracy of a single ultrasound method is not sufficient to diagnose the presence of metastases in the SLNs of mammary glands with tumors. B-mode ultrasonography through S/L ratio and ecostructure guides the diagnosis; the location of the blood vessels, their type, distribution and the resistivity index, examined by the Doppler technique, increase the confidence of the diagnosis; enhancement pattern and wash-out time evaluated by CEUS are high predictors for the presence of metastases and the lymph nodes stiffness evaluated by the elasticity scores, strengthens the diagnostic certainty. All these techniques, available for most of the veterinary practitioners, are easy to perform and the described algorithm helps in staging mammary gland tumors, guiding the appropriate therapeutic approach, thus avoiding the unjustified excision of SLNs.

Results obtained in our study support the use of the four techniques as non-invasive identification and primary assessment of sentinel lymph nodes in help selecting further interventions, determining therapeutic approach and prognosis.

## Figures and Tables

**Table 1:** Five-point elasticity scoring system.

Elasticity Score	Description	Interpretation
**1**	Total green or yellow, absent blue areas or very small blue area/s	Soft
**2**	Small scattered blue areas or total blue area <45%	Moderately soft
**3**	Large blue area/s, total blue area ≥45%	Moderately stiff
**4**	Peripheral large hard area and central small green areas suggesting central necrosis	Predominantly stiff
**5**	Hard area occupying the entire lymph node, with or without green rim	Stiff

**Table 2:** Histological classification of encountered mammary tumors.

Type of Neoplasms	Number of Neoplasms
Carcinoma in a mixed tumor	25
Carcinoma—simple tubular	19
Carcinoma—simple tubulopapillary	17
Carcinoma—solid	5
Carcinoma—complex type	3
Carcinoma—anaplastic	2
Total subjects	71

**Table 3:** Qualitative and quantitative variables evaluated by different ultrasonography methods: B-mode, Doppler technique, contrast-enhanced ultrasonography and real-time elastography.

Ultrasound Parameter *n* (%)	Unaffected Nodes (N = 42)	Metastatic/Affected Nodes (N = 54)	*p* Value
**B-MODE ULTRASONOGRAPHY OF SENTINEL LYMPH NODES**			
SA/LA ratio < 0.55	33 (78.57)	9(16.66)	<0.001
Short axis (mean ± SD) cm	0.67 ± 0.26	1.17 ± 0.53	
Long axis (mean ± SD) cm	1.33 ± 0.46	1.83 ± 0.79	
**Internal structure (Echostructure)**			
Homogeneous	36(85.71)	10(18.51)	<0.001
Inhomogeneous	6(14.28)	44(81.48)	
**Echogenicity**			
Hypoechoic	28(66.66)	37(68.51)	=0.81
Isoechoic	10(23.80)	14(25.92)	
Hyperechoic	4(9.52)	3(5.55)	
**Hillum tissue definition**			
Present	30(71.48)	29(53.70)	=0.07
Absent/invisible	12(28.57)	25(46.29)	
**Capsule (Borders)**			
Well-defined	37(88.09)	39(72.22)	0.057
Ill-defined	5 (11.90)	15(27.77)	
**COLOR DOPPLER ULTRASONOGRAPHY—VASCULAR PATTERN**			
**Localization**			
Absence of vascular signal	2(4.76)	1(1.85)	<0.001
Hilar pattern	36(85.71)	12(22.22)	
Peripheral pattern	1(2.38)	30(55.55)	
Mixed	3(7.14)	11(20.37)	
**Type and distribution**			
Ordered	36(85.71)	5(9.25)	<0.001
Chaotic	4(9.52)	48(88.88)	
NA	2(4.76)	1(1.85)	
**Intranodal vascular resistance (expressed by mean ± SD)**			
RI	0.5060 ± 0.147	0.7011 ± 0.138	<0.001
PI	0.9828 ± 0.223	1.2426 ± 0.259	
**CONTRAST-ENHANCED ULTRASONOGRAPHY (CEUS)**			
Enhancement patterns			
Intense homogeneouss	32(76.19)	6(11.11)	<0.001
Inhomogeneous	8(19.04)	44(81.48)	
No enhancement	2(4.76)	4(7.40)	
**Perfusion times (expressed by mean ± SD)**			
Wash in time/s	18.12 ± 7.15	15.34 ± 7.23	=0.07
Wash out time/s	151.48 ± 34.62	104.26 ± 39.67	<0.001
**REAL-TIME ELASTOGRAPHY—LYMPH NODE STIFFNESS ASSESSED BY ELASTICITY STIFFNESS SCORES**			
Soft (scores 1 and 2)	38(90.47)	6(11.11)	<0.001
Hard (scores 3, 4 and 5)	6(14.28)	48(88.88)	

Legend: SA—short axis; LA—long axis; NA—not applicable; RI—resistivity index; PI—pulsatility index; s—seconds.

**Table 4:** Lymph node characterization according to the elasticity score.

Lymph Nodes	1	2	3	4	5
Unaffected (N = 42)	25 (59.5)	13 (31.0)	3 (7.1)	-	1 (2.4)
Metastatic (N = 54)	2 (3.7)	4 (7.4)	14 (25.9)	19 (35.2)	15 (27.8)
*p* value	<0.001				

Note—number in parenthesis are percentages.

**Table 5:** ROC analysis. Diagnostic performance of significant parameters of gray-sale US, Doppler US, CEUS, and sonoelastography in the detection of metastatic lymph nodes.

Criterion	AUC	SE (%)	SP (%)	Statistical Sig.	Cutoff Value	CI 95%	PPV (%)	NPV (%)	Acc (%)
S/L Ratio	0.812	83.3	78.6	Good	0.550	0.724–0.899	83.33	78.57	81.25
L axis	0.689	50.0	92.9	Poor	1.860	0.583–0.794	90.00	59.09	68.75
S axis	0.798	64.8	85.7	Fair	0.854	0.711–0.885	85.37	65.45	73.96
RI	0.837	83.0	75.0	Good	0.565	0.753–0.921	81.02	77.45	80.00
PI	0.798	83.0	65.0	Fair	1.025	0.705–0.891	75.31	74.86	75.14
WOT (s)	0.818	84.0	74.4	Good	133.0	0.727–0.910	80.81	78.33	79.78
ES	0.928	88.9	90.5	Excellent	2.5	0.871–0.986	89.69	86.36	89.71

Legend: AUC—area under the curve; SE—sensitivity; SP—specificity; PPV—positive predictive value; NPV—negative predictive value; Acc—accuracy; RI—resistivity index; PI—pulsatility index; WOT—wash-out time; ES—elasticity score.

**Table 6:** Proposed algorithm for differentiating between unaffected and metastatic sentinel lymph nodes using available ultrasound techniques—grayscale US, Doppler, CEUS and elastography.

	US Parameter	Pattern	Score
**Gray scale ultrasound**			
1.	S/L ratio	<0.55	0
		≥0.55	1
2.	Echostructure	Homogeneous	0
		Inhomogeneous	1
**Doppler ultrasound**			
3.	Localization	Hilar	0
		Peripheral	1
4.	Type and distribution	Ordered	0
		Chaotic	1
5.	RI	<0.54	0
		≥0.54	1
6.	PI	<0.83	0
		≥0.83	1
Score < 3—unaffected lymph node; Score = 3 CEUS and elastography should be performed; Score ≥ 3 metastatic lymph node should be taken into account			
**CEUS**			
1.	Enhancement pattern	Intense, homogeneous	0
		Inhomogeneous ± no enhanced areas	1
2.	Wash-out time (s) peritumoral ad.	>133	0
		≤133	1
**Strain elastography**			
3.	Stiffness	Soft or moderately soft (scores 1 and 2)	0
		Predominantly/moderately stiff or stiff (scores 3,4,5)	1

Considering CEUS and elastography: Score ≤ 1—unaffected lymph node; Score ≥ 2—malignant lymph node. Considering all significant US parameters: score = 3—unaffected lymph node: score ≥ 4—malignant lymph node. Legend: S—short axis; L—long axis; RI—resistivity index; PI—pulsatility index.

## Supplementary Materials

The following are available online at https://www.mdpi.com/2076-2615/10/12/2366/s1. File 1: Demographic characteristics, histological diagnosis of mammary tumor and lymphatic drainage of involved tumoral mammary gland.

## References

[1] Abadie J., Nguyen F., Loussouarn D., Peña L., Gama A., Rieder N., Belousov A., Bemelmans I., Jaillardon L., Ibisch C. (2018). Canine invasive mammary carcinomas as models of human breast cancer. Part 2: Immunophenotypes and prognostic significance. Breast Cancer Res. Treat..

[2] Nguyen F., Peña L., Ibisch C., Loussouarn D., Gama A., Rieder N., Belousov A., Campone M., Abadie J. (2018). Canine invasive mammary carcinomas as models of human breast cancer. Part 1: Natural history and prognostic factors. Breast Cancer Res. Treat..

[3] Coleto A.F., Wilson T.M., Soares N.P., Gundim L.F., Castro I.P., Guimarães E.C., Bandarra M.B., Medeiros-Ronchi A.A. (2018). Prognostic value of occult isolated tumour cells within regional lymph nodes of dogs with malignant mammary tumours. J. Comp. Pathol..

[4] Chocteau F., Abadie J., Loussouarn D., Nguyen F. (2019). Proposal for a histological staging system of mammary carcinomas in dogs and cats. part 1: Canine mammary carcinomas. Front. Vet. Sci..

[5] Bauer T.W., Spitz F.R., Callans L.S., Alavi A., Mick R., Weinstein S.P., Bedrosian I., Fraker D.L., Bauer T.L., Czerniecki B.J. (2002). Subareolar and peritumoral injection identify similar sentinel nodes for breast cancer. Ann. Surg. Oncol..

[6] Edwina Doting M.H., Annemiek Stiekema H.M., de Vries J., Lemstra C., Hoekstra H.J., Vrieling M., Rietman L., Jager P.L. (2007). Immediate dynamic lymphoscintigraphy delivers no additional value to lymphoscintigraphy 3 hr after tracer injection in sentinel lymph node biopsy in breast cancer patients. J. Surg. Oncol..

[7] Tuttle T.M., Colbert M., Christensen R., Ose K.J., Jones T., Wetherille R., Friedman J., Swenson K., McMasters K.M. (2002). Subareolar injection of 99mTc facilitates sentinel iymph node identification. Ann. Surg. Oncol..

[8] McGregor A., Pavri S.N., Tsay C., Kim S., Narayan D. (2017). Use of indocyanine green for sentinel lymph node biopsy: Case series and methods comparison. Plast. Reconstr. Surg. Glob. Open.

[9] Vermersch C., Raia-Barjat T., Chapelle C., Lima S., Chauleur C. (2019). Randomized comparison between indocyanine green fluorescence plus 99m technetium and 99m technetium alone methods for sentinel lymph node biopsy in breast cancer. Sci. Rep..

[10] Favril S., Stock E., Hernot S., Hesta M., Polis I., Vanderperren K., de Rooster H. (2019). Sentinel lymph node mapping by near-infrared fluorescence imaging and contrast-enhanced ultrasound in healthy dogs. Vet. Comp. Oncol..

[11] Kawase K., Gayed I.W., Hunt K.K., Kuerer H.M., Akins J., Yi M., Grimes L., Babiera G.V., Ross M.I., Feig B.W. (2006). Use of lymphoscintigraphy defines lymphatic drainage patterns before sentinel lymph node biopsy for breast cancer. J. Am. Coll. Surg..

[12] Rossi F., Körner M., Suárez J., Carozzi G., Meier V.S., Roos M., Rohrer Bley C. (2018). Computed tomographic-lymphography as a complementary technique for lymph node staging in dogs with malignant tumors of various sites. Vet. Radiol. Ultrasound.

[13] Soultani C., Patsikas M.N., Karayannopoulou M., Jakovljevic S., Chryssogonidis I., Papazoglou L., Papaioannou N., Papadopoulou P., Pavlidou K., Ilia G.M. (2017). Assessment of sentinel lymph node metastasis in canine mammary gland tumors using computed tomographic indirect lymphography. Vet. Radiol. Ultrasound.

[14] Seiler S.M.F., Baumgartner C., Hirschberger J., Beer A.J., Brühschwein A., Kreutzmann N., Laberke S., Wergin M.C., Meyer-Lindenberg A., Brandl J. (2015). Comparative oncology: Evaluation of 2-deoxy-2-[18F]. Fluoro-d-glucose (FDG) Positron emission tomography/computed tomography (PET/CT) for the staging of dogs with malignant tumors. PLoS ONE.

[15] Sánchez D., Romero L., López S., Campuzano M., Ortega R., Morales A., Guadarrama M., Cesarman-Maus G., García-Pérez O., Lizano M. (2019). 18F-FDG—PET/CT in canine mammary gland tumors. Front. Vet. Sci..

[16] Suga K., Yuan Y., Ogasawara N., Okada M., Matsunaga N. (2003). Localization of breast sentinel lymph nodes by MR lymphography with a conventional gadolinium contrast agent. Acta Radiol..

[17] De Swarte M., Alexander K., Rannou B., D’Anjou M.A., Blond L., Beauchamp G. (2011). Comparison of sonographic features of benign and neoplastic deep lymph nodes in dogs. Vet. Radiol. Ultrasound.

[18] Llabrés-Díaz F.J. (2004). Ultrasonography of the medial iliac lymph nodes in the dog. Vet. Radiol. Ultrasound.

[19] Nyman H.T., O’Brien R.T. (2007). The sonographic evaluation of lymph nodes. Clin. Tech. Small Anim. Pract..

[20] Stan F., Gudea A., Baba A.I., Feier D., Badea R. (2012). Correlation between ultrasonographic features and morphological pattern after blue dye injection of normal superficial lymph nodes in carnivores. Bull. Univ. Agric. Sci. Vet. Med. Cluj Napoca Vet. Med..

[21] Cox K., Taylor-Phillips S., Sharma N., Weeks J., Mills H., Sever A., Lim A., Haigh I., Hashem M., De Silva T. (2018). Enhanced pre-operative axillary staging using intradermal microbubbles and contrast-enhanced ultrasound to detect and biopsy sentinel lymph nodes in breast cancer: A potential replacement for axillary surgery. Br. J. Radiol..

[22] Mei M., Ye L., Quan J., Huang P. (2018). Contrast-enhanced ultrasound for the differential diagnosis between benign and metastatic superficial lymph nodes: A meta-analysis. Cancer Manag. Res..

[23] Nielsen Moody A., Bull J., Culpan A.M., Munyombwe T., Sharma N., Whitaker M., Wolstenhulme S. (2017). Preoperative sentinel lymph node identification, biopsy and localisation using contrast enhanced ultrasound (CEUS) in patients with breast cancer: A systematic review and meta-analysis. Clin. Radiol..

[24] Sharma N., Cox K. (2017). Axillary nodal staging with contrast-enhanced ultrasound. Curr. Breast Cancer Rep..

[25] Liptak J.M., Boston S.E. (2019). Nonselective lymph node dissection and sentinel lymph node mapping and biopsy. Vet. Clin. North Am. Small Anim. Pract..

[26] Stan F.G. (2016). Non-invasive assessment of sentinel lymph nodes that drain the tumoral mammary glands in female dog. Bull. UASVM Vet. Med..

[27] Gelb H.R., Freeman L.J., Rohleder J.J., Snyder P.W. (2010). Feasibility of contrast-enhanced ultrasound-guided biopsy of sentinel lymph nodes in dogs. Vet. Radiol. Ultrasound.

[28] Belotta A.F., Gomes M.C., Rocha N.S., Melchert A., Giuffrida R., Silva J.P., Mamprim M.J. (2019). Sonography and sonoelastography in the detection of malignancy in superficial lymph nodes of dogs. J. Vet. Intern. Med..

[29] Seiler G.S., Griffith E. (2018). Comparisons between elastographic stiffness scores for benign versus malignant lymph nodes in dogs and cats. Vet. Radiol. Ultrasound.

[30] Silva P., Uscategui R.A.R., Maronezi M.C., Gasser B., Pavan L., Gatto I.R.H., de Almeida V.T., Vicente W.R.R., Feliciano M.A.R. (2018). Ultrasonography for lymph nodes metastasis identification in bitches with mammary neoplasms. Sci. Rep..

[31] Choi Y.J., Lee J.H., Baek J.H. (2015). Ultrasound elastography for evaluation of cervical lymph nodes. Ultrasonography.

[32] Kim H.J., Kim S.M., Kim B., La Yun B., Jang M., Ko Y., Lee S.H., Jeong H., Chang J.M., Cho N. (2018). Comparison of strain and shear wave elastography for qualitative and quantitative assessment of breast masses in the same population. Sci. Rep..

[33] Pereira C.T., Rahal S.C., De Carvalho Balieiro J.C., Ribeiro A.A.C.M. (2003). Lymphatic drainage on healthy and neoplasic mammary glands in female dogs: Can it really be altered?. J. Vet. Med. Ser. C Anat. Histol. Embryol..

[34] Patsikas M.N., Karayannopoulou M., Kaldrymidoy E., Papazoglou L.G., Papadopoulou P.L., Tzegas S.I., Tziris N.E., Kaitzis D.G., Dimitriadis A.S., Dessiris A.K. (2006). The lymph drainage of the neoplastic mammary glands in the bitch: A lymphographic study. J. Vet. Med. Ser. C Anat. Histol. Embryol..

[35] Stan F. (2012). Correlation between subareolar and peritumoral blue dye injection to identify sentinel lymph nodes in canine mammary neoplazia. Lucr. Ştiinţifice Ser. Med. Vet. Iasi.

[36] Patsikas M.N., Dessiris A. (1996). The lymph drainage of the mammary glands in the bitch: A lymphographic study. Part I: The 1st, 2nd, 4th and 5th mammary glands. Anat. Histol. Embryol..

[37] Patsikas M.N., Dessiris A. (1996). The lymph drainage of the mammary glands in the bitch: a lymphographic study. Part II: The 3rd mammary gland. Anat. Histol. Embryol..

[38] Rossi F., Fina C., Stock E., Vanderperren K., Duchateau L., Saunders J.H. (2016). Effect of sedation on contrast-enhanced ultrasonography of the spleen in healthy dogs. Vet. Radiol. Ultrasound.

[39] Stock E., Vanderperren K., Van der Vekens E., Haers H., Duchateau L., Polis I., Hesta M., Saunders J.H. (2014). The effect of anesthesia with propofol and sedation with butorphanol on quantitative contrast-enhanced ultrasonography of the healthy feline kidney. Vet. J..

[40] Du Sert N.P., Hurst V., Ahluwalia A., Alam S., Avey M.T., Baker M., Browne W.J., Clark A., Cuthill I.C., Dirnagl U. (2020). The arrive guidelines 2.0: Updated guidelines for reporting animal research. PLoS Biol..

[41] Dudea S.M., Lenghel M., Botar-Jid C., Vasilescu D., Duma M. (2012). Ultrasonography of superficial lymph nodes: benign vs. malignant. Med. Ultrason..

[42] Nyman H.T., Kristensen A.T., Skovgaard I.M., McEvoy F.J. (2005). Characterization of normal and abnormal canine superficial lymph nodes using gray-scale b-mode, color flow mapping, power, and spectral doppler ultrasonography: A multivariate study. Vet. Radiol. Ultrasound.

[43] Soler M., Dominguez E., Lucas X., Novellas R., Gomes-Coelho K.V., Espada Y., Agut A. (2016). Comparison between ultrasonographic findings of benign and malignant canine mammary gland tumours using b-mode, colour doppler, power doppler and spectral doppler. Res. Vet. Sci..

[44] Feliciano M.A.R., Uscategui R.A.R., Maronezi M.C., Simões A.P.R., Silva P., Gasser B., Pavan L., Carvalho C.F., Canola J.C., Vicente W.R.R. (2017). Ultrasonography methods for predicting malignancy in canine mammary tumors. PLoS ONE.

[45] Choi M.Y., Lee J.W., Jang K.J. (1995). Distinction between benign and malignant causes of cervical, axillary, and inguinal lymphadenopathy: Value of doppler spectral waveform analysis. Am. J. Roentgenol..

[46] Sever A.R., Mills P., Jones S.E., Mali W., Jones P.A. (2012). Sentinel node identification using microbubbles and contrast-enhanced ultrasonography. Clin. Radiol..

[47] Xie F., Zhang D., Cheng L., Yu L., Yang L., Tong F., Liu H., Wang S., Wang S. (2015). Intradermal microbubbles and contrast-enhanced ultrasound (CEUS) is a feasible approach for sentinel lymph node identification in early-stage breast cancer. World J. Surg. Oncol..

[48] Poanta L., Serban O., Pascu I., Pop S., Cosgarea M., Fodor D. (2014). The place of CEUS in distinguishing benign from malignant cervical lymph nodes: A prospective study. Med. Ultrason..

[49] Alam F., Naito K., Horiguchi J., Fukuda H., Tachikake T., Ito K. (2008). Accuracy of sonographic elastography in the differential diagnosis of enlarged cervical lymph nodes: Comparison with conventional b-mode sonography. Am. J. Roentgenol..

[50] Goldschmidt M.H., Peña L., Rasotto R., Zappulli V. (2011). Classification and grading of canine mammary tumors. Vet. Pathol..

[51] Vassallo P., Edel G., Roos N., Naguib A., Peters P.E. (1993). In-vitro high-resolution ultrasonography of benign and malignant lymph nodes. Invest. Radiol..

[52] Cassali G.D., Lavalle G.E., de Nardi A.B., Ferreira E., Bertagnolli A.C., Estrela-Lima A., Alessi A.C., Daleck C.R., Salgado B.S., Fernandes C.G. (2011). Consensus for the diagnosis, prognosis and treatment of canine mammary tumors. Braz. J. Vet. Pathol..

[53] Karin U., Sorenmo D.R., Worley M.H.G., Stephen J., Withrow D.M., Vail R.L.P. (2013). Tumors of the mammary gland. Small Animal Clinical Oncology.

[54] De Araújo M.R., Campos L.C., Ferreira E., Cassali G.D. (2015). Quantitation of the regional lymph node metastatic burden and prognosis in malignant mammary tumors of dogs. J. Vet. Intern. Med..

[55] Beserra H.E.O., Grandi F., Dufloth R.M., Pinheiro L.G.P., Miot H.A., Vexenat S.C.O.R., Rocha N.S. (2016). Metastasis of mammary carcinoma in bitches: Evaluation of the sentinel lymph node technique. Adv. Breast Cancer Res..

[56] Lorek A., Stojčev Z., Zarębski W., Kowalczyk M., Szyluk K. (2019). Analysis of postoperative complications after 303 sentinel lymph node identification procedures using the sentimag® method in breast cancer patients. Med. Sci. Monit..

[57] Luigi Solbiati V., Cioffi E.B. (1992). Ultrasonography of the neck. Radiol. Clin. N. Am..

[58] Nyman H.T., Nielsen O.L., McEvoy F.J., Lee M.H., Martinussen T., Hellmén E., Kristensen A.T. (2006). Comparison of b-mode and doppler ultrasonographic findings with histologic features of benign and malignant mammary tumors in dogs. Am. J. Vet. Res..

[59] Silver T.I., Lawson J.A., Mayer M.N. (2012). Sonographic characteristics of presumptively normal main axillary and superficial cervical lymph nodes in dogs. Am. J. Vet. Res..

[60] Salwei R.M., O’Brien R.T., Matheson J.S. (2005). Characterization of lymphomatous lymph nodes in dogs using contrast harmonic and power doppler ultrasound. Vet. Radiol. Ultrasound.

[61] Zandvliet M. (2016). Canine Lymphoma: A Review. Vet. Q..

[62] Alvarez S., Añorbe E., Alcorta P., López F., Alonso I., Cortés J. (2006). Role of sonography in the diagnosis of axillary lymph node metastases in breast cancer: A systematic review. Am. J. Roentgenol..

[63] Chen X., Li X., Fan Z., Li J., Xie Y., Wang T., Ouyang T. (2020). Ultrasound as a replacement for physical examination in clinical staging of axillary lymph nodes in breast cancer patients. Thorac. Cancer.

[64] Mayer M.N., Lawson J.A., Silver T.I. (2010). Sonographic characteristics of presumptively normal canine medial iliac and superficial inguinal lymph nodes. Vet. Radiol. Ultrasound.

[65] Ruppel M.J., Pollard R.E., Willcox J.L. (2019). Ultrasonographic characterization of cervical lymph nodes in healthy dogs. Vet. Radiol. Ultrasound.

[66] Burns G.O., Scrivani P.V., Thompson M.S., Erb H.N. (2008). Relation between age, body weight, and medial retropharyngeal lymph node size in apparently healthy dogs. Vet. Radiol. Ultrasound.

[67] Krol L., O’Brien R. (2012). Ultrasonographic assessment of abdominal lymph nodes in puppies. Vet. Radiol. Ultrasound.

[68] Ahmadi O., Mccall J.L., Stringer M.D. (2013). Does senescence affect lymph node number and morphology? a systematic review. ANZ J. Surg..

[69] Xin L., Yan Z., Zhang X., Zang Y., Ding Z., Xue H., Zhao C. (2017). Parameters for contrast-enhanced ultrasound (CEUS) of enlarged superficial lymph nodes for the evaluation of therapeutic response in lymphoma: a preliminary study. Med. Sci. Monit..

[70] Dialani V., James D.F., Slanetz P.J. (2015). A Practical approach to imaging the axilla. Insights Imaging.

[71] Ahuja A.T., Ying M., Ho S.Y., Antonio G., Lee Y.P., King A.D., Wong K.T. (2008). Ultrasound of malignant cervical lymph nodes. Cancer Imaging.

[72] Nieciecki M., Dobruch-Sobczak K., Wareluk P., Gumińska A., Białek E., Cacko M., Królicki L. (2016). Rola badania ultrasonograficznego oraz limfoscyntygrafii w diagnostyce węzłów chłonnych pachowych u pacjentek z rakiem piersi. J. Ultrason..

[73] Kinns J., Mai W. (2007). Association between malignancy and sonographic heterogeneity in canine and feline abdominal lymph nodes. Vet. Radiol. Ultrasound.

[74] Agthe P., Caine A.R., Posch B., Herrtage M.E. (2009). Ultrasonographic appearance of jejunal lymph nodes in dogs without clinical signs of gastrointestinal disease. Vet. Radiol. Ultrasound.

[75] Maxwell F., De Margerie Mellon C., Bricout M., Cauderlier E., Chapelier M., Albiter M., Bourrier P., Espié M., De Kerviler E., De Bazelaire C. (2015). Diagnostic strategy for the assessment of axillary lymph node status in breast cancer. Diagn. Interv. Imaging.

[76] Choi H.Y., Park M., Seo M., Song E., Shin S.Y., Sohn Y.M. (2017). Preoperative Axillary Lymph Node Evaluation in Breast Cancer: Current Issues and Literature Review. Ultrasound Q..

[77] Prativadi R., Dahiya N., Kamaya A., Bhatt S. (2017). Chapter 5 ultrasound characteristics of benign vs malignant cervical lymph nodes. Semin. Ultrasound CT MRI.

[78] Ying M., Bhatia K.S.S., Lee Y.P., Yuen H.Y., Ahuja A.T. (2014). Review of ultrasonography of malignant neck nodes: Greyscale, doppler, contrast enhancement and elastography. Cancer Imaging.

[79] Almerey T., Villacreses D., Li Z., Patel B., McDonough M., Gibson T., Maimone S., Gray R., McLaughlin S.A. (2019). Value of axillary ultrasound after negative axillary mri for evaluating nodal status in high-risk breast cancer. J. Am. Coll. Surg..

[80] Misra D., Panjwani S., Rai S., Misra A., Prabhat M., Gupta P., Talukder S. (2016). Diagnostic efficacy of color doppler ultrasound in evaluation of cervical lymphadenopathy. Dent. Res. J. (Isfahan).

[81] Stan F.G. (2010). Power Dopler Ultrasonography vs color dopler of the sentinel lymph mammary glands at female dog. Bull. Univ. Agric. Sci. Vet. Med. Cluj Napoca Vet. Med..

[82] Park S.H., Jeong Y.M., Cho S.H., Jung H.K., Kim S.J., Ryu H.S. (2014). imaging findings of variable axillary mass and axillary lymphadenopathy. Ultrasound Med. Biol..

[83] Ahuja A.T., Ying M., Ho S.S.Y., Metreweli C. (2001). Distribution of intranodal vessels in differentiating benign from metastatic neck nodes. Clin. Radiol..

[84] Rotim T., Kristek B., Turk T., Kretić D., Perić M., Pušeljić I., Pandurović T., Štefanić M. (2017). Measurable and unmeasurable features of ultrasound lymph node images in detection of malignant infiltration. Acta Clin. Croat..

[85] Stan F. (2015). Qualitative Morphological Assessement of Tumor Associated Lymphatic Vasculature in Mammary Gland Neoplasia of Female Dog in Relation With Sentinel Lymph Nodes Metastatic Infiltration. Sci. Work. Ser. C. Vet. Med..

[86] Apple S.K. (2016). Sentinel lymph node in breast cancer: review article from a pathologist’s point of view. J. Pathol. Transl. Med..

[87] Prieto S., Gomez-Ochoa P., De Blas I., Gascón M., Aceña C., Corda A., Sosa I., Gregori T., Couto G. (2009). Pathologic correlation of resistive and pulsatility indices in canine abdominal lymph nodes. Vet. Radiol. Ultrasound.

[88] El-Gohary Y.M., Metwally G., Saad R.S., Robinson M.J., Mesko T., Poppiti R.J. (2008). Prognostic significance of intratumoral and peritumoral lymphatic density and blood vessel density in invasive breast carcinomas. Am. J. Clin. Pathol..

[89] Goldberg B.B., Merton D.A., Liu J. (2005). Contrast-enhanced sonographic imaging of lymphatic channels and sentinel lymph nodes. J. Ultrasound Med..

[90] Goldberg B.B., Merton D.A., Liu J., Thakur M., Murphy G.F., Needleman L., Tornes A. (2004). Sentinel lymph nodes in a swine model with melanoma: contrast enhanced lymphatic US. Radiology.

[91] Platt A.M., Randolph G.J. (2013). Dendritic Cell Migration Through the Lymphatic Vasculature to Lymph Nodes.

[92] Skobe M., Detmar M. (2000). Structure, function, and molecular control of the skin lymphatic system. J. Investig. Dermatol. Symp. Proc..

[93] Sever A., Broillet A., Schneider M., Cox K., Jones S., Weeks J., Mills P., Fish D., Jones P. (2010). Dynamic visualization of lymphatic channels and sentinel lymph nodes using intradermal microbubbles and contrast-enhanced ultrasound in a swine model and patients with breast cancer. J. Ultrasound Med..

[94] Shimazu K., Ito T., Uji K., Miyake T., Aono T., Motomura K., Naoi Y., Shimomura A., Shimoda M., Kagara N. (2017). Identification of sentinel lymph nodes by contrast-enhanced ultrasonography with sonazoid in patients with breast cancer: a feasibility study in three hospitals. Cancer Med..

[95] Liu J., Liu X., He J., Gou B., Luo Y., Deng S., Wen H., Zhou L. (2019). Percutaneous contrast-enhanced ultrasound for localization and diagnosis of sentinel lymph node in early breast cancer. Sci. Rep..

[96] Zhao J., Zhang J., Zhu Q.L., Jiang Y.X., Sun Q., Zhou Y.D., Wang M.Q., Meng Z.L., Mao X.X. (2018). The value of contrast-enhanced ultrasound for sentinel lymph node identification and characterisation in pre-operative breast cancer patients: a prospective study. Eur. Radiol..

[97] Mori N., Mugikura S., Miyashita M., Kudo Y., Suzuki M., Li L., Mori Y., Takahashi S., Takase K. (2019). Perfusion contrast-enhanced ultrasound to predict early lymph-node metastasis in breast cancer. Jpn. J. Radiol..

[98] Ouyang Q., Chen L., Zhao H., Xu R., Lin Q. (2010). Detecting metastasis of lymph nodes and predicting aggressiveness in patients with breast carcinomas. J. Ultrasound Med..

[99] Zenk J., Bozzato A., Hornung J., Gottwald F., Rabe C., Gill S., Iro H. (2007). Neck lymph nodes: prediction by computer-assisted contrast medium analysis?. Ultrasound Med. Biol..

[100] Yang H.K., Burns P.N., Jang H.J., Kono Y., Khalili K., Wilson S.R., Kim T.K. (2019). Contrast-enhanced ultrasound approach to the diagnosis of focal liver lesions: the importance of washout. Ultrasonography.

[101] Piscaglia F., Nolsøe C., Dietrich C.F., Cosgrove D.O., Gilja O.H., Bachmann Nielsen M., Albrecht T., Barozzi L., Bertolotto M., Catalano O. (2012). The EFSUMB guidelines and recommendations on the clinical practice of contrast enhanced ultrasound (CEUS): Update 2011 on non-hepatic applications. Ultraschall der Medizin.

[102] Badea R., Ciobanu L. (2012). Contrast enhanced and doppler ultrasonography in the characterization of the microcirculation. expectancies and performances. Med. Ultrason..

[103] Choi M., Yoon J., Choi M. (2019). Semi-quantitative strain elastography may facilitate pre-surgical prediction of mandibular lymph nodes malignancy in dogs. J. Vet. Sci..

[104] Bhatia K.S., Cho C.C., Yuen Y.H., Rasalkar D.D., King A.D., Ahuja A.T. (2010). Real-time qualitative ultrasound elastography of cervical lymph nodes in routine clinical practice: Interobserver agreement and correlation with malignancy. Ultrasound Med. Biol..

[105] Sigrist R.M.S., Liau J., El Kaffas A., Chammas M.C., Willmann J.K. (2017). Ultrasound elastography: Review of techniques and clinical applications. Theranostics.

[106] Lo W.C., Liao L.J. (2014). Comparison of two elasticity scoring systems in the assessment of the cervical lymph nodes. J. Med. Ultrasound.

[107] Zhao Q.L., Xia X.N., Zhang Y., He J.J., Sheng W., Ruan L.T., Yin Y.M., Hou H.L. (2018). Elastosonography and two-dimensional ultrasonography in diagnosis of axillary lymph node metastasis in breast cancer. Clin. Radiol..

[108] Wojcinski S., Dupont J., Schmidt W., Cassel M., Hillemanns P. (2012). Real-time ultrasound elastography in 180 axillary lymph nodes: Elasticity distribution in healthy lymph nodes and prediction of breast cancer metastases. BMC Med. Imaging.

[109] Lenghel L.M., Bolboacǎ S.D., Botar-Jid C., Bǎciut G., Dudea S.M. (2012). The value of a new score for sonoelastographic differentiation between benign and malignant cervical lymph nodes. Med. Ultrason..

[110] Bhatia K.S.S., Lee Y.Y.P., Yuen E.H.Y., Ahuja A.T. (2013). Ultrasound elastography in the head and neck. part ii. accuracy for malignancy. Cancer Imaging.

[111] Bhatia K.S.S., Lee Y.Y.P., Yuen E.H.Y., Ahuja A.T. (2013). Ultrasound elastography in the head and neck. part, i. basic principles and practical aspects. Cancer Imaging.

[112] Dietrich C.F., Barr R.G., Farrokh A., Dighe M., Hocke M., Jenssen C., Dong Y., Saftoiu A., Havre R.F. (2016). 1 introduction to elastography. Elastography.

[113] Jung J.W., Je H., Lee S.K., Jang Y., Choi J. (2020). Two-Dimensional shear wave elastography of normal soft tissue organs in adult beagle dogs; interobserver agreement and sources of variability. Front. Bioeng. Biotechnol..

[114] Wang M., Zhou W., Zhao Y., Xia T., Zha X., Ding Q., Liu X., Zhao Y., Ling L., Chen L. (2012). A novel finding of sentinel lymphatic channels in early stage breast cancer patients: which may influence detection rate and false-negative rate of sentinel lymph node biopsy. PLoS ONE.

[115] Wang Y., Zhou W., Li C., Gong H., Li C., Yang N., Zha X., Chen L., Xia T., Liu X. (2017). variation of sentinel lymphatic channels (SLCs) and sentinel lymph nodes (SLNs) assessed by contrast-enhanced ultrasound (CEUS) in breast cancer patients. World J. Surg. Oncol..

[116] Guo J., Yang H., Wang S., Cao Y., Liu M., Xie F., Liu P., Zhou B., Tong F., Cheng L. (2017). Comparison of sentinel lymph node biopsy guided by indocyanine green, blue dye, and their combination in breast cancer patients: A prospective cohort study. World J. Surg. Oncol..

[117] Joseph F.J., Van Oepen A., Friebe M. (2017). Breast sentinel lymph node biopsy with imaging towards minimally invasive surgery. Biomed. Tech..

[118] Takemoto N., Koyanagi A., Yamamoto H., Shimura K., Fujii R. (2014). Comparison of the indocyanine green dye method versus the combined method of indigo carmine blue dye with indocyanine green fluorescence imaging for sentinel lymph node biopsy in patients with stage i or ii breast cancer. Ann. Oncol..

[119] Siddique M., Nawaz M.K., Bashir H. (2018). The usefulness of SPECT/CT in sentinel node mapping of early stage breast cancer patients showing negative or equivocal findings on planar scintigraphy. Asia Ocean. J. Nucl. Med. Biol..

[120] Taumberger N., Pernthaler B., Schwarz T., Bjelic-Radisic V., Pristauz G., Aigner R.M., Tamussino K. (2020). lymphoscintigraphy for sentinel lymph node biopsy in breast cancer: do we need a delayed image?. Breast Care.

[121] Niu G., Chen X. (2015). Lymphatic imaging: Focus on imaging probes. Theranostics.

[122] Ahmed M., Purushotham A.D., Horgan K., Klaase J.M., Douek M. (2015). Meta-Analysis of superficial versus deep injection of radioactive tracer and blue dye for lymphatic mapping and detection of sentinel lymph nodes in breast cancer. Br. J. Surg..

[123] Zada A., Peek M.C.L., Ahmed M., Anninga B., Baker R., Kusakabe M., Sekino M., Klaase J.M., Haken B., Douek M. (2016). Meta-analysis of sentinel lymph node biopsy in breast cancer using the magnetic technique. Br. J. Surg..

[124] Karakatsanis A., Michael P., Lone C. (2016). The nordic sentimag trial: A comparison of super paramagnetic iron oxide (spio) nanoparticles versus tc 99 and patent blue in the detection of sentinel node (sn) in patients with breast cancer and a meta-analysis of earlier studies. Breast cancer Res. Treat..

[125] Martonos C., Gudea A., Dezdrobitu C., Damian A., Crisan M., Bud I., Pentea M. (2019). Morphological description of sentinel lymphatic channels in canine mammary tumours. Proceedings of the Multidisciplinary Conference on Sustainable Development.

[126] Zhao Y.C., Ni X.J., Li Y., Dai M., Yuan Z.X., Zhu Y.Y., Luo C.Y. (2012). Peritumoral lymphangiogenesis induced by vascular endothelial growth factor c and d promotes lymph node metastasis in breast cancer patients. World, J. Surg. Oncol..

[127] Widodo I., Dwianingsih E.K., Utoro T., Anwar S.L., Aryandono T. (2018). Prognostic value of lymphangiogenesis determinants in luminal and non-luminal breast carcinomas. Asian Pacific J. Cancer Prev..

