# Supplementary material for: Ultrasonographic Algorithm for the Assessment of Sentinel Lymph Nodes That Drain the Mammary Carcinomas in Female Dogs
Source: Animals (Basel). 2020 Dec 10;10(12):2366. doi: 10.3390/ani10122366 (PMC7763578; doi:10.3390/ani10122366)
Supplement: Supplementary file 1 [file animals-10-02366-s001.pdf]

Supplementary file 1

Demographic characteristics, histological diagnosis of mammary tumor and lymphatic drainage of involved tumoral mammary gland													
**No.**	**Breed**	**Age**	**Histological diagnosis of mammary tumor**	**Tumor location**				**Sentinel lymph nodes**				**Histological diagnosis of lymph nodes**	
				**T_1**	**A_1 A_2I**	**A_2I**	**I**	**Acc ax**	**Pr ax**	**St cr**	**Ing sup**	**Benign**	**Metastatic**
**1**	German Shepherds	8,9	**CMT**	+					+	+		+	
**2**	Rottweiler	10,6	**CMT**	+					+				+
**3**	Yorkshire terriers	11,3	**CMT**	+				+				+	
**4**	Mixed breed	12,0	**CMT**		+			+			+	**+ (Acc ax)**	**+ (Ing sup)**
**5**	Mixed breed	10,4	**CMT**		+				+		+		++
**6**	Cocker Spaniels	9,5	**CMT**		+				+		+		++
**7**	German Shepherds	8,9	**CMT**		+						+		+
**8**	Rottweiler	9,6	**CMT**		+						+	+	
**9**	Doberman Pinschers	8,8	**CMT**		+						+		+
**10**	Mixed breed	10,5	**CMT**		+						+		+
**11**	Yorkshire terriers	12,1	**CMT**		+						+		+
**12**	German Shepherds	8,7	**CMT**		+						+	+	
**13**	Doberman Pinschers	9,8	**CMT**		+						+	+	
**14**	Mixed breed	10,9	**CMT**		+						+	+	
**15**	Mixed breed	11,6	**CMT**			+					+		+
**16**	Dachshund	14,1	**CMT**			+					+		+
**17**	Mixed breed	12,4	**CMT**			+					+	+	
**18**	German Shepherds	9,8	**CMT**			+					+		+
**19**	Doberman Pinschers	8,9	**CMT**				+				++	++	
**20**	Doberman Pinschers	8,0	**CMT**				+				++	++	
**21**	Cocker Spaniels	11,5	**CMT**				+				++		++
**22**	Mixed breed	11,2	**CMT**				+				+	+	
**23**	Doberman Pinschers	10,9	**CMT**				+				+		+
**24**	Mixed breed	13,6	**CMT**				+				+		+
**25**	Mixed breed	12,5	**CMT**				+				+		+
**26**	Doberman Pinschers	9,1	**CS–T**			+					+	+	
**27**	German Shepherds	8,1	**CS–T**			+					++	++	
**28**	Mixed breed	8,6	**CS–T**			+					+		+
**29**	Mixed breed	8,9	**CS–T**			+					++	++	
**30**	Mixed breed	9,7	**CS–T**			+					+		+
**31**	German Shepherds	11,2	**CS–T**			+					+	+	
**32**	Mixed breed	8,5	**CS–T**			+					+	+	
**33**	Cocker Spaniels	10,0	**CS–T**			+					+		+
**34**	Doberman Pinschers	9,3	**CS–T**			+					+	+	
**35**	German Shepherds	9,1	**CS–T**			+					+		+
**36**	Mixed breed	9,8	**CS–T**				+				++		
**37**	Mixed breed	11,2	**CS–T**				+				+	+	
**38**	Doberman Pinschers	9,7	**CS–T**				+				++		++
**39**	Cocker Spaniels	11,2	**CS–T**				+				+		+
**40**	Dachshund	14,1	**CS–T**				+				+	+	
**41**	Mixed breed	10,1	**CS–T**				+				+		+
**42**	Mixed breed	9,5	**CS–T**				+				+	+	
**43**	Rottweiler	8,9	**CS–T**				+				+		+
**44**	Mixed breed	11,6	**CS–T**				+				++	++	
**45**	Mixed breed	12,5	**CS–TP**	+				+					+
**46**	Mixed breed	13,6	**CS–TP**	+					+	+		+	
**47**	Yorkshire terriers	12,5	**CS–TP**	+					+				+
**48**	Mixed breed	11,5	**CS–TP**	+					+			+	
**49**	Mixed breed	9,7	**CS–TP**		+			+			+		++
**50**	Mixed breed	8,9	**CS–TP**		+				+		+	++	
**51**	German Shepherds	8,0	**CS–TP**		+				+		+		++

**52**	Rottweiler	9,0	**CS–TP**		+				+		+		++
**53**	Doberman Pinschers	10,8	**CS–TP**		+						+	+	
**54**	Mixed breed	11,7	**CS–TP**				+				++		++
**55**	Mixed breed	14,2	**CS–TP**				+				++	++	
**56**	Yorkshire terriers	11,9	**CS–TP**				+				++	++	
**57**	Mixed breed	10,6	**CS–TP**				+				+		+
**58**	Rottweiler	9,2	**CS–P**				+				+	+	
**59**	Mixed breed	9,1	**CS–TP**				+				+		+
**60**	Mixed breed	10,5	**CS–TP**				+				+		+
**61**	Mixed breed	8,9	**CS–TP**				+				+	+	
**62**	Rottweiler	8,7	**CS**	+				+					+
**63**	Yorkshire terriers	12,5	**CS**	+					+			+	
**64**	Mixed breed	14,5	**CS**		+			+			+		++
**65**	Rottweiler	10,3	**CS**				+				++	++	
**66**	Cocker Spaniels	11,7	**CS**				+				++		++
**67**	German Shepherds	8,9	**CC**				+				++	++	
**68**	Mixed breed	12,0	**CC**				+				++		++
**69**	Dachshund	14,6	**CC**				+				+		+
**70**	Doberman Pinschers	10,6	**CA**				+				++		++
**71**	German Shepherds	9,2	**CA**				+				++		++
			**Total**	9	**17**	14	**31**	6	11	**2**	**79**		++
			**TOTAL**	**71 subjects**				**17 axillary ln**		**2 st ct ln**	**79 sup ing ln**	**42 benign**	**54 metastatic**
								**96 evaluated SLN**				**96 evaluated SLN**	

Legend: Histological diagnosis of mammary tumor according to Goldschmidt *et al.*, 2011 [49]. CMT—carcinoma in a mixed tumor; CS–T—carcinoma simple tubular; C–TP—carcinoma simple tubulopapillary; CS—carcinoma solid; CC—carcinoma complex type; CA—carcinoma anaplastic. T_1—cranial thoracic mammary gland; A_1—cranial abdominal mammary gland; A_2—caudal abdominal mammary gland; I—inguinal mammary gland; Pr ax—proper axillary lymph node; Acc ax—accessory axillary lymph node; St cr—cranial sternal lymph node; Ing sup—superficial inguinal lymph nodes.
